# Ultra-long-acting in-situ forming implants with cabotegravir protect female macaques against rectal SHIV infection

**DOI:** 10.1038/s41467-023-36330-5

**Published:** 2023-02-09

**Authors:** Isabella C. Young, Ivana Massud, Mackenzie L. Cottrell, Roopali Shrivastava, Panita Maturavongsadit, Alka Prasher, Andres Wong-Sam, Chuong Dinh, Tiancheng Edwards, Victoria Mrotz, James Mitchell, Josilene Nascimento Seixas, Aryani Pallerla, Allison Thorson, Amanda Schauer, Craig Sykes, Gabriela De la Cruz, Stephanie A. Montgomery, Angela D. M. Kashuba, Walid Heneine, Charles W. Dobard, Martina Kovarova, J. Victor Garcia, J. Gerardo Garcίa-Lerma, S. Rahima Benhabbour

**Affiliations:** 1 Division of Pharmacoengineering and Molecular Pharmaceutics, Eshelman School of Pharmacy, University of North Carolina at Chapel Hill, Chapel Hill, NC USA; 2 Laboratory Branch, Division of HIV Prevention, National Center for HIV/AIDS, Viral Hepatitis, STD, and TB Prevention, Centers for Disease Control and Prevention, Atlanta, GA USA; 3 Division of Pharmacotherapy and Experimental Therapeutics, Eshelman School of Pharmacy, University of North Carolina at Chapel Hill, Chapel Hill, NC USA; 4 Joint Department of Biomedical Engineering, North Carolina State University and The University of North Carolina at Chapel Hill, Chapel Hill, NC USA; 5 Comparative Medicine Branch, Division of Scientific Resources, National Center for Emerging and Zoonotic Infection Diseases, Centers for Disease Control and Prevention, Atlanta, GA USA; 6 Infectious Diseases Pathology Branch, Division of High-Consequence Pathogens and Pathology, National Center for Emerging and Zoonotic Infection Diseases, Centers for Disease Control and Prevention, Atlanta, GA USA; 7 Department of Psychology and Neuroscience, University of North Carolina at Chapel Hill, Chapel Hill, NC USA; 8 Pathology Services Core, Lineberger Comprehensive Cancer Center, University of North Carolina School of Medicine, Chapel Hill, NC USA; 9 International Center for the Advancement of Translational Science, Division of Infectious Diseases, Center for AIDS Research, University of North Carolina at Chapel Hill, Chapel Hill, NC USA

**Keywords:** Drug delivery, HIV infections, Antiviral agents, Rhesus macaque

## Abstract

Ultra-long-acting delivery platforms for HIV pre-exposure prophylaxis (PrEP) may increase adherence and maximize public health benefit. We report on an injectable, biodegradable, and removable in-situ forming implant (ISFI) that is administered subcutaneously and can release the integrase inhibitor cabotegravir (CAB) above protective benchmarks for more than 6 months. CAB ISFIs are well-tolerated in female mice and female macaques showing no signs of toxicity or chronic inflammation. In macaques, median plasma CAB concentrations exceed established PrEP protection benchmarks within 3 weeks and confer complete protection against repeated rectal SHIV challenges. Implant removal via a small incision in 2 macaques at week 12 results in a 7- to 48-fold decrease in plasma CAB levels within 72 hours. Modeling to translate CAB ISFI dosing suggests that a 3 mL injection would exceed protective benchmarks in humans for over 5 months post administration. Our results support the clinical advancement of CAB ISFIs for ultra-long-acting PrEP in humans.

## Abstract (web summary)

In this study, the authors developed an ultra-long-acting injectable, biodegradable, and removable in-situ forming implant delivering cabotegravir (CAB ISFI). CAB ISFI was well tolerated and protected against multiple rectal SHIV challenges in female macaques.

## Introduction

As of 2021, 38.4 million people are currently living with HIV and 40.1 million people have died from AIDS-related illnesses worldwide since the start of the epidemic^1^. Pre-exposure prophylaxis (PrEP) with daily oral regimens containing emtricitabine (FTC) in combination with tenofovir disoproxil fumarate (TDF) or tenofovir alafenamide has been highly effective in preventing HIV acquisition when taken with high adherence^2,3^. Although high adherence will result in high PrEP efficacy, maintaining high adherence to daily oral PrEP remains a major challenge^4–6^. Low levels of adherence and subsequent infection can also lead to the development of drug-resistant viruses^7^. Furthermore, low levels of adherence are particularly seen among young sub-Saharan African women due to high stigma, low product acceptability, and/or the inability to disclose product use to their sexual partners^5^. Mitigating adherence issues in sub-Saharan Africa, specifically in women, is key to the success of PrEP since Sub-Saharan Africa accounts for over 60% of all new HIV infection worldwide^1,5^. To this end, the pipeline for HIV prevention options is moving towards developing long-acting (LA) PrEP products that do not require frequent dosing and may overcome some of the adherence challenges associated with daily oral PrEP. In fact, studies have shown that long-acting products have greater adherence and acceptability to daily oral regimens^5,8–11^, particularly in populations where HIV prevalence is highest. Thus, increased PrEP adherence and acceptability from long-acting HIV PrEP can ultimately reduce HIV transmission rates.

An injectable long-acting formulation of the integrase inhibitor cabotegravir (CAB LA) was approved in late 2021 by the FDA for PrEP in men and women^12^. The approval of CAB LA followed results from the HPTN 083 and 084 trials showing that CAB LA was safe and more effective than daily oral FTC/TDF, likely reflecting the adherence advantage of long-acting PrEP^13–15^. The studies also defined the plasma CAB concentrations needed for protection to be four times above the protein-adjusted 90% inhibitory concentration (4× PA-IC_90,_ 664 ng/mL^16^). CAB LA is administered in 3 mL intramuscular injections each month for 2 months initially and bi-monthly thereafter. Although CAB LA presents a major advance in HIV PrEP and treatment, the large injection volumes (3 mL/injection), injection site reactions^14,17^, and the inability to be self-administered or removed to terminate treatment if required are challenges yet to be addressed. Additionally, due to its long terminal half-life (>40 days)^16^ and inability to be removed after administration, CAB LA demonstrates a long pharmacologic tail with low but detectable levels of CAB remaining in the plasma for over 15 months after discontinuation of treatment^18,19^. These suboptimal levels of CAB can result in breakthrough infections and development of drug-resistant HIV, thus requiring supplemental oral PrEP to cover the tail to prevent future infections^19^. To overcome these limitations, efforts are now shifting to the development of removable, self-administered (e.g., subcutaneous administration) ultra-long-acting CAB formulations that sustain protective plasma CAB levels through extended dosing intervals such as every 6 months or longer. Such formulations may facilitate large-scale implementation and maximize cost-effectiveness and public health benefit in both resource-poor and -rich countries.

In situ forming implants (ISFIs) may provide desirable properties for an ultra-long-acting CAB formulation including long dosing intervals, small injection volumes, and retrievability^20,21^. ISFIs consist of a hydrophobic and biodegradable polymer (e.g., poly(lactic-co-glycolic acid) (PLGA)), biocompatible water-miscible organic solvents (e.g., N-methyl-2-pyrrolidone (NMP) or dimethyl sulfoxide (DMSO)), and active pharmaceutical ingredients (APIs)^22–24^ that are co-formulated to generate a homogenous and syringeable liquid solution or suspension. Upon injection into the intramuscular or subcutaneous space, the water-miscible organic solvent diffuses into the aqueous environment, resulting in a phase inversion generating a solid or semi-solid depot comprising the API entrapped within the precipitated polymer matrix^23–26^. APIs are released from the depot via diffusion through the polymer matrix and via polymer bulk degradation over time.

ISFIs formulated with PLGA and NMP have been extensively studied for the long-acting release of dolutegravir (DTG), an HIV integrase inhibitor analog of CAB^20,21^. In NOD scid common gamma chain knockout mice and bone marrow–liver–thymus (BLT) mice, ISFIs released DTG for over 11 months at levels that were 10–100-fold above the PA-IC_90_. These levels were associated with high protection from multiple high-dose vaginal HIV challenges and suppression of viremia in infected mice^20,21^. Furthermore, although implant removal is not required due to PLGA’s biodegradable nature, the study showed the ability to easily retrieve the depot via a small skin incision. After removal, DTG levels in plasma fell below the PA-IC_90_ within 24 hours and below the limit of detection after 7 days^20^. Collectively these observations highlighted the potential for ISFIs as a novel ultra-long-acting delivery system and supported the extended evaluation of ISFIs delivering CAB.

Herein, we developed and characterized a CAB ISFI formulation with high drug loading and that is amenable for subcutaneous administration in small volumes. We defined stability, microstructure, injectability, and release kinetics in vitro and in vivo. We show that this formulation is safe in female mice and non-human primates and can release CAB for 6–11 months at levels that are above established benchmarks for PrEP protection in macaques and humans. We associate the extended release of CAB from the ISFI with long-lasting protection against SHIV infection in a macaque model of PrEP that predicted clinical efficacy of CAB LA and other approved oral PrEP regimens. Our study identifies a promising platform for the extended release of CAB at levels that are known to be associated with PrEP protection in humans.

## Results

### Optimization of CAB ISFI and in vitro drug release

Many factors influence drug release kinetics from ISFIs, including polymer type and molecular weight (MW), solvent, polymer-to-solvent ratio, miscibility of the drug and polymer with the solvent, and drug physiochemical properties^20,24,27^. Here, we investigated the effect of solvents, drug loading, polymer molecular weight, and polymer-to-solvent ratios to develop an ISFI with maximum CAB loading and high in vitro release rates. First, we investigated CAB saturation solubility in various solvents to determine the ideal solvent system for the formulation to achieve maximum drug loading (Supplementary Table 1). NMP and DMSO are water-miscible organic solvents commonly used in ISFI formulations^24^, thus these solvents were tested in various weight ratios. The addition of excipients such as Tween 20, Pluronics, and Gelucire was also investigated as they can improve the aqueous solubility of poorly insoluble drugs^28^. Based on CAB solubility in these various solvents, a 1:1 weight ratio (w/w) of NMP:DMSO (referred to as “solvent”) demonstrated the highest CAB solubility (saturation solubility of 167.12 ± 12.04 mg/mL) and was therefore used to formulate CAB as a stable ISFI suspension above this saturation solubility.

During optimization and building on our prior work, all CAB ISFI formulations were generated using an FDA-approved biodegradable polymer consisting of 50:50 PLGA, which enables ultra-long release of antiretrovirals from ISFIs^20,21,29^ and has a favorable degradation profile including eliciting complete degradation within a few months^30,31^. This degradation profile is ideal to ensure complete polymer degradation prior to subsequent ISFI injections to prevent polymer accumulation. Furthermore, all formulations consisted of 50:50 PLGA with low MWs of 10 kDa or 27 kDa, as PLGAs with lower MWs can accommodate higher drug loading, elicit greater drug release, and have lower viscosity to ensure syringeability^31^ compared to PLGA with higher MWs when used in ISFI systems^24,32,33^.

**Fig. 1:**
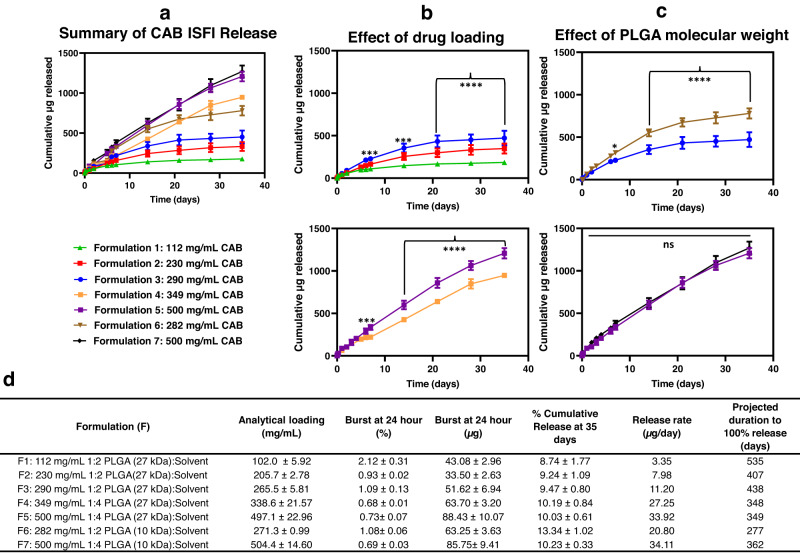
CAB ISFI cumulative in vitro release kinetics. **a** Cumulative release of CAB ISFI formulations. **b** Effect of drug loading on cumulative CAB release. **c** Effect of PLGA molecular weight on cumulative CAB release. All in vitro release studies were done in phosphate buffer saline (PBS, pH 7.4 with 2% Solutol) at 37 °C. Data presented as average ± standard deviation for *n* = 3 samples. Source data are provided as a Source Data file. Statistical analysis: two-way ANOVA with Tukey’s multiple comparison test comparing amount of drug release with respect to formulation and timepoint in **b** and **c**. *p < 0.05, ***p < 0.001, ****p < 0.0001, and ns (not significant) when *p* > 0.05. **d** Summary table of release kinetics for CAB ISFI formulations. Solvent = 1:1 (w/w) NMP:DMSO.

**Fig. 2:**
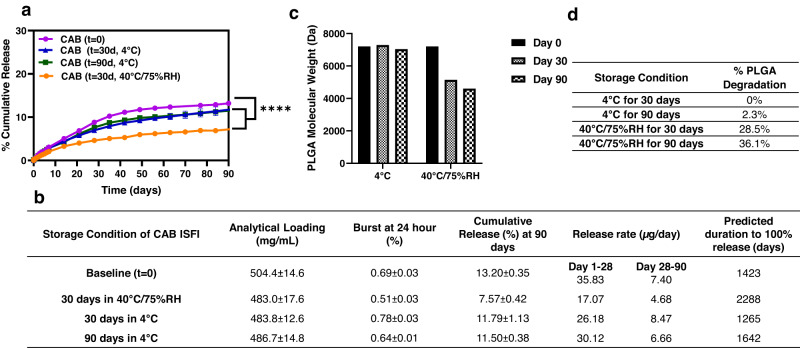
CAB ISFI stability and post-storage in vitro release kinetics. **a** Cumulative in vitro release kinetics of CAB ISFI (500 mg/mL CAB (1:4 w/w PLGA (10 kDa):solvent)) at baseline (*t* = 0), 30 days, and 90 days post-storage at 4 °C and 40 °C/75% RH. All in vitro release studies were done in phosphate buffer saline (PBS, pH 7.4 with 2% Solutol) at 37 °C. Data presented as average ± standard deviation for *n* = 3 samples. Statistical analysis: two-way ANOVA with Tukey’s multiple comparisons comparing CAB release with respect to formulation and timepoint. By day 90 post-storage in vitro release, all storage formulations elicited CAB release significantly different and slower than baseline (*p* < 0.0001) **b** Summary table of CAB ISFI post-storage in vitro release kinetics from **a**. **c** Effect of storage conditions on PLGA weight average MW of placebo formulations measured by gel permeation chromatography (GPC) analysis. **d** Summary table of PLGA degradation in placebo formulation after 30 and 90 days at 4 °C and 40 °C/75% RH. Solvent = 1:1 w/w NMP:DMSO. Source data are provided as a Source Data file.

Cumulative in vitro release studies of seven CAB ISFI formulations were evaluated for 35 days (Fig. 1a) to investigate the effect of drug loading, PLGA MW, and polymer-to-solvent ratios to determine the optimal formulation with the highest drug loading and release rates (Fig. 1 and Supplementary Fig. 1). Results showed that CAB release rates increased with (1) increasing drug loading (Fig. 1b), (2) increasing the amount of solvent, and (3) decreasing the MW of PLGA (Fig. 1c). All formulations exhibited very low burst release (<3% release within 24 hours), sustained release for over one month, and projected to release for more than 6 months in vitro (Fig. 1d). ISFIs containing 349 mg/mL CAB (1:4 w/w PLGA (27 kDa):solvent) (Formulation 4), 500 mg/mL CAB (1:4 w/w PLGA (27 kDa):solvent) (Formulation 5), and 500 mg/mL CAB (1:4 w/w PLGA (10 kDa):solvent) (Formulation 7) exhibited zero-order release kinetics for the first 35 days. Additionally, CAB release was significantly different (*p* < 0.05) when varying the drug loading (Fig. 1b) and PLGA MW (Fig. 1c). Notably, ISFIs containing 500 mg/mL CAB (1:4 w/w PLGA (10 kDa):solvent) (Formulation 7) and 500 mg/mL (1:4 w/w PLGA (27 kDa):solvent) (Formulation 5) exhibited comparable release kinetics (*p* > 0.05), despite differences in PLGA MW.

Similar release rates between Formulation 5 and 7 is likely attributed to the considerably high drug loading in the formulations (500 mg/mL CAB), which drives the CAB release profile, regardless of differences in PLGA MW. In both formulations, the PLGA to CAB ratio (w/w) is 1:3.5 w/w (41.2% CAB and 11.8% PLGA). Thus, the difference of PLGA MW in Formulations 5 and 7 did not produce as large of an impact in CAB release compared to other formulations with different PLGA MWs, such as Formulation 3 and 6 (PLGA to CAB ratio of 1:0.75 w/w).

Based on similar and promising drug release profiles from Formulation 5 and 7, in vitro release for these formulations was extended to 180 days (Supplementary Fig. 2a). Although we observed higher in vitro release of CAB from Formulation 5 after ~2 months compared to Formulation 7, a pilot mouse study showed no difference in CAB concentrations in plasma between the two formulations for up to 90 days (Supplementary Fig. 2b). Formulation 7 was selected for subsequent studies due to its lower PLGA MW (10 kDa). The lower PLGA MW elicited lower viscosity (Supplementary Table 2) ensuring syringeability. Moreover, the 10 kDa PLGA (Formulation 7) is expected to degrade faster compared to the 27 kDa PLGA (Formulation 5) and was selected to ensure complete polymer degradation and mitigate polymer buildup upon subsequent doses^24,32,33^.

Ultimately, the 500 mg/mL CAB (1:4 w/w PLGA (10 kDa):solvent) ISFI (Formulation 7) (referred to as CAB ISFI) was selected as the optimized formulation owing to its high drug loading and high in vitro release rate and was further characterized in terms of depot microstructure. It is known that drug release kinetics are influenced by depot microstructure, which is further influenced by the polymer, solvent, drug physiochemical properties, and miscibility of drug with the solvent^20,24^. As shown in Supplementary Fig. 3, CAB ISFI microstructure was qualitatively analyzed with scanning electron microscopy (SEM) and formed a densely packed depot with CAB crystals, and the microstructure remained unaltered with no apparent pore formation in the ISFI microstructure over 90 days incubation in release media in vitro. Furthermore, we performed scanning electron microscopy energy dispersive X-ray (SEM EDX) analysis on CAB ISFI and placebo ISFI (CAB-free) to confirm CAB crystals seen in SEM images (Supplementary Fig 4). SEM EDX results demonstrated the absence of crystals within the placebo ISFI and confirmed crystals were composed of CAB in the CAB ISFI from the elemental analysis (presence of fluorine groups, Supplementary Fig. 4). Ultimately, these results corroborate the in vitro and in vivo release kinetics of CAB over 90 days.

### Stability and post-storage in vitro release studies

To determine the shelf-life of the optimized CAB ISFI, we performed stability studies at two storage conditions (40 °C/75% relative humidity (RH) and 4 °C) for 30 and 90 days followed by analysis of post-storage release in vitro. Stability was determined based on physical appearance (color, viscosity, phase separation) of ISFI formulation and drug stability and concentration after storage.

After 30 days at 4 °C or 40 °C/75% RH and 90 days at 4 °C, the CAB ISFI formulation showed no visual difference in physical appearance (color, syringeability, phase separation). Drug concentration was comparable to the initial concentration (*t* = 0) (<5% difference) with no drug degradation peaks observed by high performance liquid chromatography (HPLC) (Supplementary Fig. 5a). The formulation was no longer injectable nor homogenous after 90 days at 40 °C/75% RH (Supplementary Fig. 5b), which precluded the analysis of post-storage release kinetics.

Figure 2a and b show post-storage in vitro release kinetics obtained after 30 days at 4 °C or 40 °C/75% RH and after 90 days at 4 °C. Regardless of the storage condition, CAB release after storage was slower and significantly different (*p* < 0.05) compared to the formulation at baseline (*t* = 0). Specifically, CAB in vitro release when stored at 40 °C/75% RH and 4 °C became significantly different compared to baseline after 7 days (*p* = 0.0042) and 21 days (*p* = 0.0004), respectively. Ultimately, all formulations were significantly different (*p* < 0.0001) compared to baseline after 90 days of in vitro release (Fig. 2a).

This decrease in release kinetics was likely influenced by PLGA hydrolysis under stability storage conditions. PLGA degradants can crystallize during hydrolysis^24^, resulting in a more crystalline polymer network which can slow drug release. To confirm degradation, gel permeation chromatography (GPC) analysis of the placebo ISFI formulation (1:4 w/w PLGA (10 kDa):solvent) demonstrated a 2.3% decrease in MW when stored at 4 °C and a 36.1% decrease in MW when stored at 40 °C/75% RH for 90 days (Fig. 2c and d) relative to the initial PLGA MW (*t* = 0). Additionally, the pH of the placebo formulation was measured before and after storage. At baseline and after 90 days in storage at 4 °C, the pH of the placebo formulation was neutral (pH = 6–7) whereas the pH became more acidic (pH = 4) after 90 days of storage at 40 °C/75% RH, further confirming PLGA degradation to its acid byproducts, specifically after storage in 40 °C/75% RH. Overall, CAB ISFI was more stable at storage in 4 °C as reflected by minimum PLGA degradation (2.3%) and the ability to retain formulation injectability and homogeneity after 90 days.

### In vivo safety studies in BALB/c mice

**Fig. 3:**
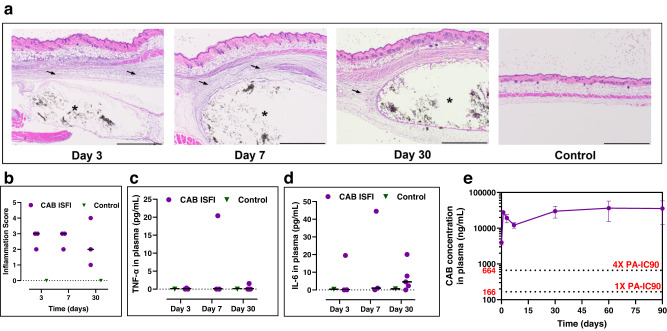
In vivo safety evaluation and in vivo drug release of CAB ISFIs in BALB/c mice. **a** Local inflammation of excised depots and surrounding subcutaneous tissues collected at day 3, 7, and 30 post-injection (*n* = 3/timepoint for CAB ISFI treated mice and *n* = 1/timepoint for control (no injection) mice) and stained with H&E. Asterisks indicate CAB implant. Arrows indicate infiltrated immune cells and areas of inflammation. All scale bars represent 1 mm. **b** Inflammatory score of subcutaneous tissue surrounding the depot evaluated using a light microscope and scored blindly by a certified pathologist. Black bars represent the median inflammation score at each timepoint (*n* = 3 per timepoint). Inflammation scoring: 0: inflammatory cells present within expected limits; 1: minimal inflammation, few increased, scattered immune cells present; 2: mild inflammation, small clusters of immune cells to thin or localized tracks of inflammation or mild increase of the number of cells diffusely surrounding the depot; 3, moderate, thicker or multiple tracks of inflammation or moderate numbers of cells diffusely surrounding the depot; 4, severe, coalescing tracks of inflammation large enough to replace normal tissue architecture or severe numbers of cells diffusely surrounding the depot; 5, marked, inflammation present that is replacing expansive areas of normal tissue architecture. **c** Concentration of TNF-α (pg/mL) in plasma quantified by ELISA at day 3 (*n* = 3), 7 (*n* = 3), and 30 (*n* = 5) post-injection. **d** Concentration of IL-6 (pg/mL) in plasma quantified by ELISA at day 3 (*n* = 3), 7 (*n* = 3), and 30 (*n* = 5) post-injection. **e** CAB concentration (average ± standard deviation) in plasma (1215 mg/kg) for 90 days (*n* = 6 per timepoint). 1× and 4× PA-IC_90_ values are indicated with dotted lines for CAB (166 ng/mL and 664 ng/mL, respectively). Plasma samples of individual mice are shown in Supplementary Fig. 7. Source data are provided as a Source Data file.

In vivo safety study of CAB ISFI was conducted with female BALB/c mice to assess local and systemic inflammation post-injection compared to control mice which did not receive any treatment or injection. Results from the study showed that the CAB ISFI was well-tolerated, and mice did not show any signs of overt toxicity, behavioral changes, or weight loss (Supplementary Fig. 6). Histopathological analysis of excised implant and surrounding subcutaneous tissue demonstrated that the CAB ISFI exhibited mild to moderate local inflammation shown by infiltrated immune cells around the depot (Fig. 3a). At day 3 and 7, the median skin microscopic inflammation score was 3 (moderate inflammation) likely due to the initial immune response to the injection and decreased by day 30 in 2 out of the 3 mice tested (Fig. 3b).

Systemic inflammation was assessed by enzyme-linked immunosorbent assay (ELISA) to quantify TNF-α and IL-6 proinflammatory cytokines in plasma. Results showed no systemic acute or chronic inflammation. TNF-α ranged between 0 and 20 pg/mL and was comparable to the no injection control group (*p* = 0.5521) (Fig. 3c). IL-6 levels in plasma ranged between 0 and 45 pg/mL (Fig. 3d) and levels were comparable to those seen in the no injection control group [*p* = 0.4188 (2-way ANOVA with multiple comparisons)]. Variability in inflammation scores or proinflammatory cytokines levels can be attributed to interindividual variability, or hormone-cycle variability in mice^34^. Overall, these results demonstrated that CAB ISFIs were generally well-tolerated and considered safe with no overt signs of toxicity or chronic inflammation.

### In vivo pharmacokinetic studies in BALB/c mice

Pharmacokinetic (PK) studies were carried out in female BALB/c mice over 90 days to assess in vivo drug release kinetics of CAB ISFI. CAB plasma concentrations were quantified using a high-performance liquid chromatography-tandem mass spectrometry LC/MS-MS method and were plotted over 90 days (Fig. 3e). Furthermore, we assessed CAB release kinetics against three mathematical models (zero order, first order, and diffusion-controlled^35^). We determined that the observed CAB in vivo release in mice best fits a zero-order model (zero order model *R*^2^ = 0.97; first order model *R*^2^ = 0.87; diffusion-controlled model R^2^ = 0.86) over the 90-day PK study duration. Moreover, average CAB concentration in plasma was between 6-53-fold greater than the 4× PA-IC_90_ (664 ng/mL^16^) between all mice during the entire 90 day study (Fig. 3e and Supplementary Fig. 7).

### Assessment of CAB ISFI injectability

Based on the promising safety and PK data in BALB/c mice, the CAB ISFI formulation was selected for evaluation of safety, PK and efficacy in rhesus macaques. However, since the injection volume for macaques (two 1 mL injections) would be much higher than for mice (50 µL), it was essential to ensure formulation injectability of large volumes in vitro prior to scaling to the macaque studies. To assess injectability of the optimized CAB ISFI formulation, we utilized polyacrylamide hydrogels^36^. Polyacrylamide hydrogels have been shown to mimic the mechanical properties of in vivo subcutaneous tissue at the injection site, and elicit better correlation to in vivo release for ISFIs rather than standard in vitro release methods by direct injection into a PBS bath^36,37^. Assessing injectability was essential for the CAB ISFI formulation due to its fast phase inversion property upon injection, which is attributed to the organic solvents’ high miscibility with water and low PLGA MW^24^. If the phase inversion is too quick, the formulation could solidify between the syringe and needle and block the flow of injection.

As such, we investigated the injectability of several placebo formulations with varying polymer to solvent ratios and PLGA MW as well as the optimized CAB ISFI formulation (1:4 w/w PLGA (10 kDa):solvent). Injectability of formulations into polyacrylamide hydrogels was investigated with 16 gauge (G), 18 G, and 19 G needles with 1 mL injection volume (Supplementary Table 3). As shown in Supplementary Table 3, a 1 mL injection into the hydrogel matrix with a 19 G needle of the 1:4 w/w PLGA (10 kDa):solvent placebo or CAB ISFI formulation was not successful due to rapid phase inversion and fast depot formation leading to obstruction of formulation flow through the needle. This was likely attributed to the combination of low MW PLGA (10 kDa) and high amounts of solvent (1:4 w/w PLGA:solvent). On the other hand, 1 mL of placebo ISFIs prepared with higher PLGA MW (27 kDa) or lower amounts of solvent (1:3 and 1:2 w/w PLGA:solvent) were successfully injected into the hydrogel matrix. In addition, 1 mL injection of the optimized CAB ISFI (500 mg/mL 1:4 PLGA (10 kDa):solvent) into the hydrogel matrix was successfully achieved with an 18 G or 16 G needle. Based on these results, a 16 G needle was used to easily administer CAB ISFI formulation in macaque studies.

### CAB release from ISFIs in rhesus macaques

**Fig. 4:**
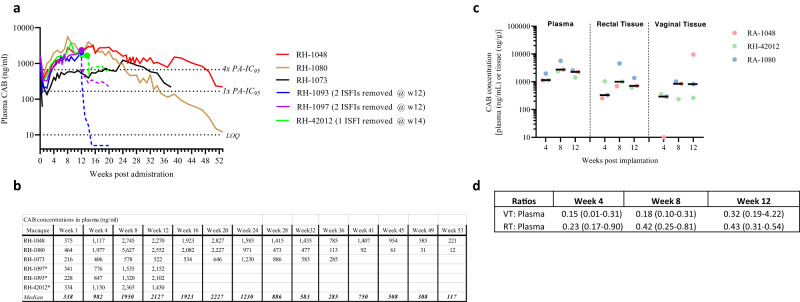
Cabotegravir concentrations in plasma and tissues in rhesus macaques treated with two CAB ISFIs. **a** Longitudinal assessment of CAB concentrations in plasma. The two CAB ISFIs were removed from macaques RH-1097 and 1093 (blue and purple solid circles) at week 12 and one of the two CAB ISFIs was removed from macaque RH-42012 at week 14 (green solid circle). Dotted lines indicate CAB levels post removal. **b** Asterisk (*) indicates animals with ISFIs removed at weeks 12–14. Data after implant removal is not included in the calculation of medians. **c** CAB levels detected in plasma, rectal tissues, and vaginal tissues from three macaques (RH-42012, RA-1048, and RA1080) with CAB ISFIs. The samples were collected from the same animals at three different time points post implantation (week 4, 8, and 12). Black bars represent the median. **d** Ratio of CAB concentrations in vaginal tissues (VT) and rectal tissues (RT) relative to plasma.

We administered CAB ISFI to six female rhesus macaques. All the animals received two separate 1 mL injections (total of 1000 mg of CAB) with the exception of one macaque (RH-1080) which received a 1.0 mL and 0.5 ml injection of ISFI or a total of 750 mg of CAB. On a per weight basis, animals received between 72.8 and 143.9 mg/kg of CAB (median = 113.8 mg/kg). Macaque RH-42012 was a SHIV-infected and otherwise healthy animal from a separate study and was included for PK purposes only. Figure 4a shows longitudinal concentrations of CAB in plasma. Overall, all the macaques achieved plasma CAB concentrations above the 4× PA-IC_90_ by week 4 with the exception of RH-1073, which achieved benchmark concentrations at week 24 (1230 ng/ml). Median (range) plasma concentrations of CAB at weeks 4, 8, and 12 were 982 [406–1977], 1950 [578–5627] and 2127 [522–2552] ng/mL, respectively, or about 1.5- to 3.2-fold above the 4× PA-IC_90_. Removal of the two CAB ISFIs in macaques RH-1097 and RH-1093 at week 12 resulted in a 7- and 48-fold reduction in plasma CAB levels at 72 h and about 10–100-fold decline at 2 weeks post removal, respectively (Fig. 4a). Likewise, removal of one of the two ISFIs that remained palpable in macaque RH-42012 at week 14 resulted in ~2-fold decline in plasma CAB within a week (1550–765 ng/mL); CAB concentrations remained above the 4× PA-IC_90_ for an additional 3 weeks. In the remaining three animals with intact CAB ISFIs, the median plasma CAB concentrations at weeks 16, 20, 24, and 28 were 1923 [534–2082], 2,227 [646–2827], 1230 [971–1585], and 886 [473–1415] ng/mL, respectively, or 1.3- to 3.4-fold above the 4× PA-IC_90_ (Fig. 4b). Notably, the plasma CAB levels in one animal (RH-1048) remained above the 4× PA-IC_90_ at week 47 (838 ng/mL). Overall, these results demonstrate that two 1 mL injections of the optimized CAB ISFI formulation can release CAB in macaques at levels above the threshold for protection for up to 6–11 months.

CAB concentrations were also measured in vaginal and rectal tissues of three macaques (RH-42012, RA-1048, and RA-1080) at weeks 4, 8, and 12. Figure 4c shows that CAB was consistently detected in both tissues, with the only exception of macaque RH-1048 which had undetectable CAB in vaginal tissues at week 4. Median CAB concentrations in rectal tissues increased ~3-fold between week 4 and week 8 (333–1004 ng/g, respectively) and slightly declined by week 12 (713 ng/g). Median CAB concentrations in vaginal tissues also increased about 2.8-fold from weeks 4 to 8 (293–849 ng/g, respectively) and remained at 823 ng/g at week 12. Tissue to plasma ratios (Fig. 4d) remained stable overtime and were similar in the vagina [median = 0.18 (0.15–0.32)] and the rectal compartment [median = 0.42 (0.23–0.43)].

### Efficacy of CAB ISFI against rectal SHIV infection

**Fig. 5:**
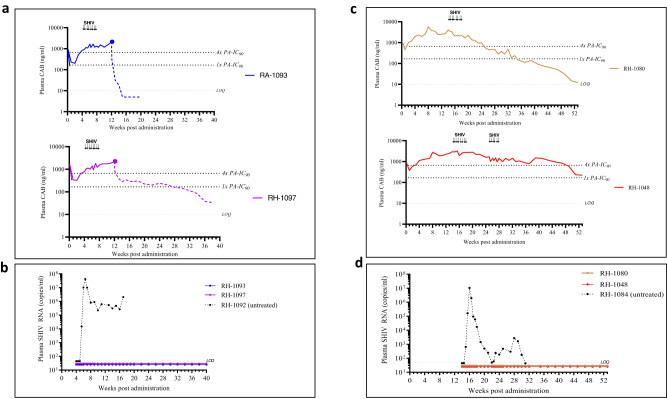
Efficacy of CAB ISFI against rectal SHIV infection in rhesus macaques. **a** Short-term protection by CAB ISFIs. Two CAB treated (RH-1093 and RH-1097) and one untreated macaque (RH-1092) were exposed rectally to SHIV between weeks 4 and 8 post implantation. Each animal received rectal SHIV challenges twice per week for up to 4 weeks (total of eight exposures). Arrows indicate SHIV exposures. ISFIs were surgically removed at week 12 (blue and purple solid circles) **b** Plasma SHIV RNA levels detected by real-time PCR in animals during challenge period and 32-week follow-up period. **c** Long-term protection by CAB ISFI. Two CAB treated (RH-1080 and RH-1048) and one untreated macaque (RH-1084) received rectal SHIV challenges twice per week between weeks 14 and 18 post implantation (up to 8 exposures). Animal RH-1048 received six additional SHIV challenges between weeks 25 and 28 (total of 14 exposures). Arrows indicate SHIV exposures. **d** Plasma SHIV RNA levels detected by real-time PCR in animals during the challenge period and the 24- to 36-week follow-up period.

To investigate if CAB delivered from ISFIs could confer rectal protection, we performed a series of SHIV challenge experiments at different times after implantation. We first evaluated short-term protection in two macaques (RH-1093 and RH-1097) challenged twice-weekly between weeks 4 and 8 (total of eight challenges per animal) (Fig. 5a). Both animals were protected against SHIV infection as opposed to an untreated real time control (RH-1092) that was infected after a single SHIV exposure. Long-term protection was evaluated in two additional macaques (RH-1048 and RH-1080) that were exposed twice per week to SHIV between weeks 14 and 18 (8 challenges per animal) (Fig. 5b). One animal (RH-1048) that maintained plasma CAB levels above 4× PA-IC_90_ received an additional six SHIV challenges between weeks 25 and 28. The two CAB treated animals were protected from infection while an untreated real time control (RH-1084) was infected after a single SHIV exposure (Fig. 5b). Overall, a single ISFI treatment completely protected 4 macaques during a cumulative of 38 rectal SHIV exposures that spanned a period of 27 weeks.

### Safety and tolerability of CAB ISFI in rhesus macaques

**Fig. 6:**
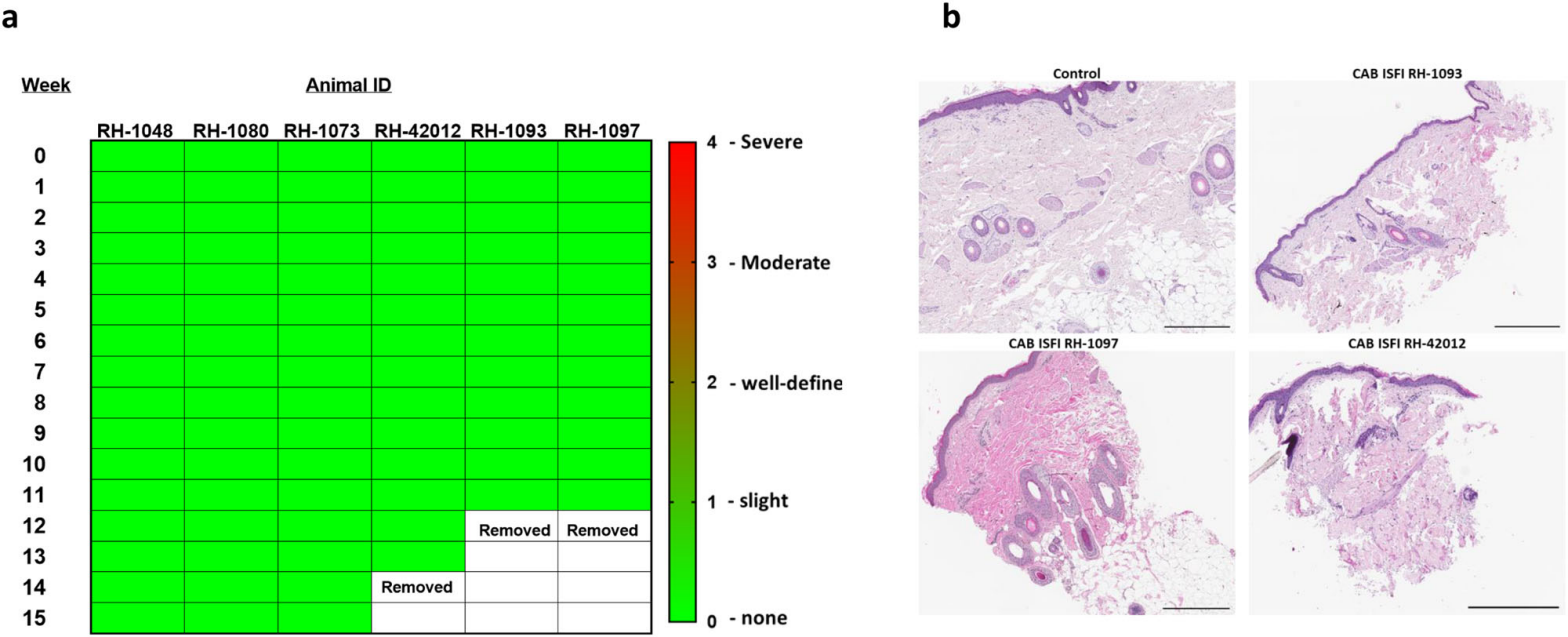
Safety and tolerability of CAB ISFI implants in rhesus macaques. **a** Heatmap of local skin reactions at the implant site in the six ISFI-treated animals. Local skin reactions were scored using a Draize scale (0-none to 4-severe). **b** Histopathology of skin. One skin biopsy per animal was collected at the implantation from treated animals RH-1093, RH-1097, RH-42012 (*n* = 3) and an untreated control (*n* = 1). All scale bars represent 1 mm (H&E, original magnification ×4).

To assess safety and tolerability, we examined the area surrounding the implants each week for up to 12 weeks or until the implant was removed. This assessment included the six macaques that received two injections for a cumulative analysis of 12 implantation sites and a total of 144 clinical observations. Based on the Draize scale, all implantation sites were unremarkable and showed no signs of local skin reactions during the 12-week study period (Fig. 6a and Supplementary Fig 8). A semiquantitative histopathological assessment was done on 3 mm surgical punch dermal biopsy collected at the implant site from three macaques during implant removal (12–14 weeks post implantation). For comparison, a 3 mm surgical punch biopsy was collected from an animal that did not receive a CAB ISFI injection. Skins biopsies had no to minimal lymphoplasmacytic infiltrates present among animals, including the control, and no evidence of infection or foreign material (residual implant) present in any of the tissue sections (Fig. 6b and Supplementary Table 4).

### Residual drug quantification and biodegradation in BALB/c mice and rhesus macaques

**Fig. 7:**
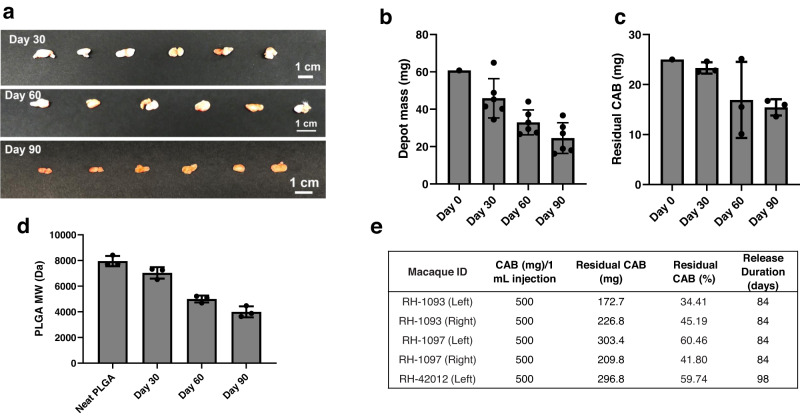
CAB ISFI biodegradation and residual drug quantification in BALB/c mice and rhesus macaques. **a** Image of CAB ISFIs retrieved from BALB/c mice 30-, 60-, and 90-days post-injection. **b** CAB ISFI masses 30-, 60-, and 90-days post-injection in mice (average ± standard deviation; *n* = 6/timepoint) compared to initial ISFI mass (day 0) from a 50 µL injection volume (60.75 mg). Day 0 masses were calculated based on a 50 µL injection volume and using the density of the formulation (1.215 g/mL) to determine approximate mass at injection. **c** Quantification of residual CAB in ISFIs 30-, 60-, and 90-days post-injection in mice (average ± standard deviation of *n* = 3 samples) compared to initial dose (25 mg in 50 µL injection). **d** Decrease in PLGA molecular weight in CAB ISFIs 30-, 60-, and 90-days post-injection in mice (average ± standard deviation of *n* = 3 samples) compared to neat PLGA (10 kDa). **e** Residual drug quantification of implants (injected in the left and right upper back location) retrieved from three rhesus macaques 84- and 98-days post-injection. Source data are provided as a Source Data file.

To assess retrievability of implants post in vivo studies, we removed CAB ISFIs from mice at days 30 (*n* = 6), 60 (*n* = 6), and 90 (*n* = 6) post administration by making a small skin incision at euthanasia. ISFIs were successfully removed from all animals with no fibrotic tissue surrounding the depot (Fig. 7a). Depots removed at day 30 (*n* = 6), 60 (*n* = 6), and 90 (*n* = 6) were further processed to evaluate polymer degradation (*n* = 3/timepoint) and residual CAB concentrations (*n* = 3/timepoint). After 90 days in mice, results from these analyses showed a 59.5 ± 13.4% loss in depot mass (Fig. 7b) and 49.7 ± 5.4% PLGA molecular weight decrease (Fig. 7c). Importantly, there was 61.6 ± 6.5% of CAB remaining in the implants retrieved from mice at day 90 demonstrating that CAB release from the ISFI can be sustained beyond 90 days (Fig. 7d).

Furthermore, both CAB ISFIs were removed from two macaques (RH-1097 and RH-1093) at day 84 and one ISFI removed from a third macaque (RH-42012) at day 98 post-injection. As shown in Fig. 7e, there was an average of 48.32 ± 11.44% CAB remaining per implant after depot removal.

### Translation of CAB ISFI dosing in macaques to clinical dosing in humans

**Fig. 8:**
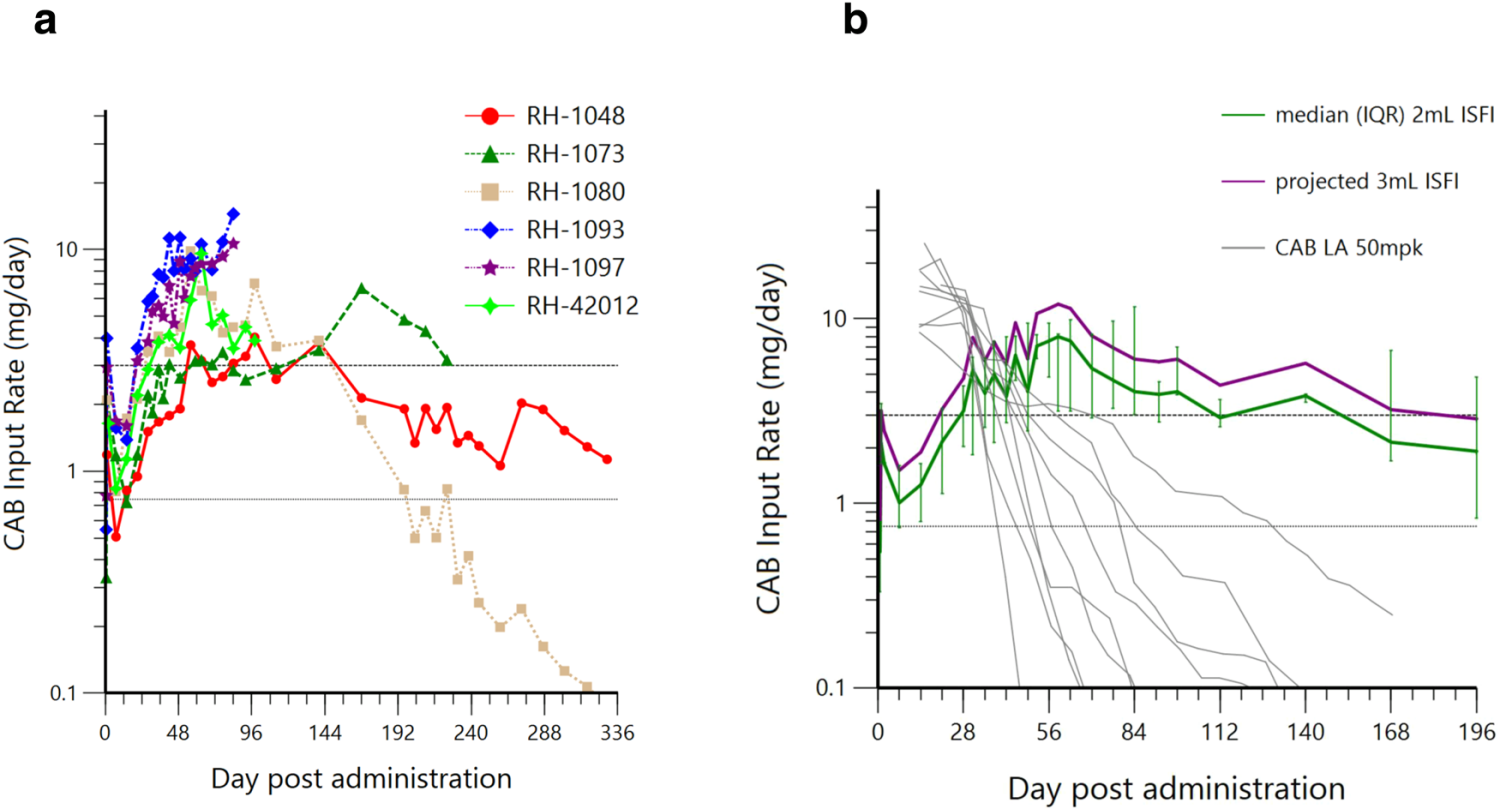
CAB input rate (mg/day) from macaque studies and modeling for clinical translation. **a** CAB ISFI input rate is estimated by multiplying observed plasma concentrations with each respective animals’ clearance as determined by their extravascular PK profile. Dashed and dotted reference lines denote input rates of 3 and 0.75 mg/day which are predicted to achieve plasma concentrations in humans above the 4× PA-IC_90_ and PA-IC_90_, respectively. **b** Median (±IQR) CAB ISFI input rates of **a** observed in this study (green line) are overlaid on median rates projected for a 3 mL injection volume (assuming input rate increases proportionally with volume; purple line) and on those estimated for *n* = 9 reference macaques dosed with 50 mpk (mg per kg) intramuscular CAB LA at 7 and 1 days prior to PK sampling^38^.

Individual CAB clearance rates were estimated (Supplementary Fig. 9) based on the extravascular PK profile for our six macaques dosed with 1.5–2 mL subcutaneous CAB ISFI [median (IQR) = 15.9 (9.1–25.2) mL/(h*kg)] and for nine historical reference macaques dosed with 50 mg/kg intramuscular CAB LA at 7 and 1 days before PK sampling [median (IQR) = 12.4 (11.7–14.3) ml/(h*kg)]^38^. Input rates for these two long-acting formulations were derived by multiplying observed plasma concentration by respective animals’ estimated clearance rate corrected for weight at time of administration in our six ISFI treated macaques or assumed weight of 8 kg in historical reference macaques (Fig. 8). Target input rates for effective clinical dosing were estimated using mean parameters from a published CAB population PK model (CL = 151 mL/h, V2 = 5270 mL, *Q* = 507 mL/h, and V3 = 2430 mL)^39^ to simulate human plasma concentrations at various IV infusion rates. Input rates of 3 and 0.75 mg/day achieved plasma concentrations above the 4× PA-IC_90_ and 1× PA-IC_90_, respectively. The median CAB ISFI input rates observed in our macaque study exceeded this 3 mg/day clinical efficacy threshold by day 28 out to day 140 post-administration. Assuming input rate increases proportionally with injection volume, a 3 mL CAB ISFI injection would achieve this threshold by day 21 out to day 168 or 5.6 months post administration. In comparison to the macaques dosed with two 50 mg/kg intramuscular CAB LA injections 6 days apart, CAB ISFIs maintained rates above the predicted protective threshold for a median of 97 extra days (Fig. 8b).

## Discussion

We report on an ultra-long-acting CAB ISFI that extends dosing intervals with small injection volumes, provides durable protection against rectal SHIV infection, and allows product retrievability. We demonstrate CAB release above established PrEP protection benchmarks for up to 6–11 months in macaques and used these data to estimate clinical exposures. We found that a 2 mL or 3 mL injection of our formulation will sustain plasma concentrations in humans above the 4× PA-IC_90_ for over 4 months or 5 months, respectively.

CAB ISFIs remained injectable, showed no drug degradation, and maintained a homogeneous CAB concentration when stored for up to 30 days in 40 °C/75% RH and up to 90 days in 4 °C, demonstrating the stability of the formulation. Although post-storage in vitro release studies caused slower CAB release at both storage conditions, there was only a ~3% difference in CAB cumulative release between baseline and after 90 days stored at 4 °C. We also performed ISFI injectability studies in polyacrylamide hydrogels to understand if fast phase inversion properties of ISFIs resulting in potential implant solidification could be a limitation associated with administration of large (≥1 mL) injection volumes. We demonstrated that a needle gauge of 18 G or 16 G can be used for injection volumes ≥1 mL. In BALB/c mice, plasma CAB concentrations were up to 53-fold greater than the 4× PA-IC_90_ for 90 days. In rhesus macaques, CAB ISFIs maintained plasma CAB concentrations above the 4× PA-IC_90_ for 6-11 months, which is unlike previously reported long-acting CAB formulations that documented much shorter periods of plasma CAB levels above protective becnhmarks^40–43^. Our in vivo safety studies in female BALB/c mice and female rhesus macaques showed that CAB ISFIs were well-tolerated with no significant levels of local or systemic inflammation. Furthermore, palpable implants were still retrievable in mice and macaques up to 90 days post-injection, demonstrating the ability to be easily removed in case of adverse events, which may eliminate the need to cover a long pharmacologic tail with oral PrEP as is the case with the current CAB LA formulation. We also noted approximately 60% of CAB remaining in the implant in mice and between 30 and 60% CAB remaining in each implant in macaques after retrieval at 84-90 days post-injection demonstrating that CAB ISFIs have the potential to release for much longer periods of time.

In rhesus macaques, a 50 mg/kg dose of injectable CAB LA provides plasma CAB concentrations above the established PrEP protection benchmark for about 6 weeks^44^. Here, we show that two 1-mL CAB ISFIs injections that contain only 1.5 to 2.9-fold more CAB on a per kg basis can sustain plasma CAB levels above this benchmark for 6–11 months. In rectal and vaginal tissues, the concentrations of CAB were also within the range of those seen in macaques treated with CAB LA and about 2-4 fold higher than those achieved in humans with the clinical 600 mg CAB LA dose, although a potential limitation is that our analysis only included the first 12 weeks post-implantation^44,45^. Additionally, ultra-long-acting CAB ISFI has notable advantages over other long-acting CAB formulations that are under preclinical development. Karunakaran et al. reported on a long-acting hydrophilic poly(ether-urethane) radiopaque CAB implants that achieved plasma levels in macaques above the 1× PA-IC_90_ for 12 weeks, but fell below the 4× PA-IC_90_ after ~2 weeks post-implantation with six implants per macaque^41^. Each implant had ~274 mg of CAB and released an average of 348 µg CAB/day in vivo^41^. Zhou et al. and Kulkarni et al. reported on an injectable nanoformulated CAB prodrug that demonstrated long-acting release of CAB in macaques (45 mg CAB equivalents/kg, ~2 mL intramuscular injection volume) for one year, however, CAB plasma concentrations fell below the 4× PA-IC_90_ and PA-IC_90_ after ~60 days^42^ and 200 days^43^, respectively. The injectable and biodegradable CAB ISFI described in our study demonstrated plasma CAB levels above the 4× PA-IC_90_ for 6–11 months in macaques and showed 100% protection against multiple rectal SHIV challenges with only two 1 mL subcutaneous injections.

Our results showing 100% protection in macaques over several months also represent to our knowledge the longest documented PrEP activity seen with a single CAB administration. We limited the SHIV challenges to periods when plasma CAB levels were above the 4× PA-IC_90_ since this concentration represents a well-established PrEP protection benchmark in macaques and humans. The complete protection observed despite a cumulative of 38 rectal SHIV exposures was thus not totally unexpected. It will be important to further define the duration of PrEP activity from CAB ISFIs since levels above the 1× PA-IC_90_ have also been associated with significant (>95%) protection against rectal SHIV challenges in macaques^40,46^. This analysis may also inform on potential reductions in CAB ISFI doses or injection volumes and on potential extension of protection beyond the predicted 5.6 months with 3 mL injection in humans. However, the documentation of rare breakthrough infections in humans who received CAB LA and maintained target levels may argue against lowering CAB dosing^47,48^.

The analysis of plasma CAB concentrations in macaques following implant removal after 3 months also provided important information about the retrievability of ISFIs. In two of the macaques, removal of the two implants resulted in a rapid 10- to 100-fold decline in plasma CAB levels within 2 weeks, although some residual implant material was evident in one of the animals as indicated by the persistent detection of CAB in plasma at levels above the PA-IC_90_. In another macaque, removal of one of the two implants resulted in a rapid 2-fold decline in plasma CAB levels within a week. In all instances, about half of the CAB dose remained in the recovered ISFI suggesting that CAB ISFIs have the potential to release for much longer periods of time. Future studies are needed to define the window of removability and to understand time to depletion of CAB from ISFIs. Although the proposed CAB ISFI has demonstrated promising potential for HIV PrEP, there are a few limitations to this formulation. For example, although we did not observe any signs of toxicity or adverse events from our mouse safety study, our results are limited due to the low sample size of the study (*n* = 3). Thus, future studies will include performing a comprehensive safety study in mice with a larger sample size and assessing long-term safety up to 180 days. Another limitation is that there is an initial decline of CAB concentration in macaques during the first two weeks post-injection with potential loss of PrEP activity as levels fall below the 4× PA-IC_90_. This period of low release, if confirmed in humans, would translate to recommending alternative HIV prevention modalities for 28 days after the first CAB ISFI injection with a 2 mL injection volume or 21 days with a 3 mL injection. Current CDC PrEP guidelines recommend alternative HIV prevention modalities for 7 to 20 days after initiating daily oral PrEP depending on potential route of transmission^49^. Another potential limitation is that the formulation includes organic solvents, which could cause toxicity at high amounts. The median lethal dose (LD_50_) and the no-observable-effect-level (NOEL) of NMP is 3600 mg/kg and 257 mg/kg, respectively in mice when administered intravenously^50^. Alternatively, the LD_50_ and NOEL of DMSO is 4000 mg/kg and 3300 mg/kg respectively in non-human primates and the LD_50_ in mice is 14,000 mg/kg when administered into the bloodstream^51^. In our study, ~300 mg/mL per solvent was administered to mice (700 mg/kg per solvent) and macaques (71 mg/kg per solvent) and did not show any signs of chronic or overt toxicity. Although the ISFIs are well below the LD_50_ and NOEL limits for DMSO and NMP in macaques, future studies should include efforts to decrease the amount of solvent to reduce potential toxicity concerns.

Additional studies to address these limitations and to further advance this study include (1) increasing the burst release of the CAB ISFI, dose administered, or implementing an oral lead-in dose to ensure concentrations in plasma remain above the 4× PA-IC_90_ during the first month after injection (2) determining the time to completion of CAB release and complete polymer degradation to assess the complete PK profile in vivo, and (3) incorporate barium sulfate to generate a radiopaque implant for X-ray monitoring to facilitate retrievability if needed and investigate implant migration. Moreover, we have demonstrated the ability to load multiple ARVs in a single ISFI achieving ultra-long-acting release kinetics in mice^20^. Thus, additional future studies may include the addition of another ARV to the current proposed CAB ISFI formulation for HIV treatment applications.

Taken together, these results highlight the promise of CAB ISFIs as an ultra-long-acting platform to deliver PrEP and support its clinical advancement into humans. The observed preclinical safety and the extended pharmacokinetic bioequivalence to CAB LA is very promising which if demonstrated clinically may result in expedited pathway for regulatory approval possibly without the need for efficacy trials. We anticipate CAB ISFIs to be highly desirable with high adherence if advanced to a clinical setting. Studies have shown that long-acting injectables elicit the greatest user acceptability and adherence in areas where HIV prevalence is highest compared to other potential dosage forms (e.g., daily oral pill and vaginal rings)^5,10^ due to low dosing frequency, familiarity, ease of use, and discretion. In addition, the proposed formulation could be self-administered since it is injected subcutaneously, which could further increase user acceptability and ease of access. Overall, ultra-long-acting CAB ISFI has the potential to be administered two to three times-a-year in a clinical setting and can advance the HIV landscape and expand preventative options around the world.

## Methods

### Materials

50:50 poly(DL-lactide-co-glycolide, PLGA) was purchased from LACTEL (Birmingham, AL; Cat. No. B6010-1P, Lot# A17–142, molecular weight 27.2 kDa, inherent viscosity 0.26-0.54 Dl/g; Cat. No. B6017-1G, Lot# A15-108, molecular weight 10 kDa, inherent viscosity 0.15-0.25 Dl/g). N-methyl-2-pyrrolidone (NMP, USP) was received from ASHLAND (Wilmington, DE, Product Code 851263, 100% NMP). Dimethyl sulfoxide (DMSO, ≥99.7%) was purchased from Fisher Scientific (Waltham, MA). Solutol-HS 15, phosphate buffered saline (0.01 M PBS, pH 7.4), and HPLC grade acetonitrile (ACN) and water were purchased from Sigma Aldrich (St. Louis, MO). Gelucire 44/14 (Cat. No. 1356950) was purchased from Sigma Aldrich (St. Louis, MO). Tween 20 was purchased from Fisher Scientific (Hampton, NJ; Cat. No. BP337-100). Tetrahydrofuran (THF) was purchased from Sigma Aldrich (St. Louis, Mo; Cat. No. SHBF9530V). 98.5% acrylamide (AM) and ammonium persulfate (APS) were purchased by Acros Organics (Carlsbad, CA; AM Cat. No. 16435000, APS Cat. No. 40116-1000), and 2% bis-acrylamide solution and N, N, N′, N′-tetramethyl ethylenediamine (TEMED) were purchased from Fisher Scientific (Hampton, NJ; bis-acrylamide Cat. No. BP150-20, TEMED Cat. No. BP1404-250). High purity (≥99%) CAB was purchased from Selleckchem (Houston, TX; Cat. No. S7766) and sterile filtered DMSO (≥99.7%) was purchased from Sigma-Aldrich (St. Louis, MO; Lot# RNBH5297).

### High performance liquid chromatography (HPLC)

A reverse-phase HPLC analysis was carried out on an Agilent 1260 HPLC system (Agilent Technologies, Santa Clara, CA, USA) with a Diode Array Detector, and LC pump with autosampler. The stationary phase utilized for CAB analysis was an Inertsil ODS-3 column (5 μm, 4.6 × 150 mm 100 Å, [GL Sciences, Torrance, CA]) maintained at 40 °C. Chromatographic separation was achieved by gradient elution using a mobile phase consisting of 0.1% trifluoroacetic acid in water and ACN (H_2_O/ACN 95:5 v/v). The flow rate was 1.0 mL/min, and the total run time was 25 min for each 25 mL injection. CAB was analyzed at 254 nm wavelength and had a retention time of 11.4 minutes. The calibration range for the assay was 0.48-250 µg/mL. Agilent OpenLab software (version C.01.08) was used for HPLC data collection.

### CAB saturation solubility

The saturation concentration of CAB in NMP, DMSO, various weight ratios (w/w) of NMP:DMSO (1:1, 9:1, and 2:8 w/w), 9:1 NMP:Gelucire 44/14 (w/w), 9:1 w/w (NMP:DMSO):Tween 20 (Supplementary Table 1) to determine optimal solvent in the ISFI system for maximum CAB loading. CAB saturation solubility was also measured in release media (PBS with 2% Solutol) to ensure sink conditions (below 1/5 of the maximum solubility^52^) during in vitro release studies (Supplementary Table 1). The addition of Solutol in release media has shown to increase the solubility of hydrophobic drugs in release media ensuring sink conditions are maintained throughout in vitro release studies^20^. CAB saturation solubility increased by 4.3-fold with the addition of 2% Solutol in PBS compared to PBS alone.

To determine saturation solubility of CAB in the following solvents shown in Supplementary Table 1, 100 mg of CAB was weighed into individual vials and 100 mg of each respective solvent was added. The mixture was stirred at 37 °C for 24 h. The samples were centrifuged for 30 min at 16,000 × *g* (Eppendorf Centrifuge 5415R, USA) to remove excess undissolved drug. Sample aliquots (*n* = 3) were collected from the saturated supernatant and diluted with ACN^20^. Drug concentration in the saturated aliquots was determined by HPLC analysis.

### Preparation of CAB ISFI formulations

50:50 poly(DL-lactide-co-glycolide) (PLGA) (27 kDa or 10 kDa molecular weight) was mixed with 1:1 weight ratio (w/w) NMP:DMSO at a 1:2 or 1:4 w/w in a 7 mL scintillation vial and dissolved by mixing at room temperature to make a homogeneous placebo formulation. Cabotegravir (CAB; 100-400 mg/g) was subsequently added to the respective polymer/solvent solution and allowed to solubilize the drug and produce a stable ISFI solution or suspension at its target drug loading. To ensure complete drug dissolution in the placebo formulation and homogeneity of the drug solution or suspension, sample aliquots (1–2 mg, *n* = 4) were collected from the drug formulation and dissolved in ACN. Drug concentration was quantified by HPLC analysis.

For in vivo studies, ISFI formulations were prepared under aseptic conditions in a biosafety cabinet. All solvents and ISFI placebo formulations were sterile filtered (Sterile Puradisc 13 mm Nylon Syringe Filter, 0.2 µm; Cat# 6786-1302). CAB was subsequently added to the sterile filtered polymer/solvent solution and solubilized to produce a stable ISFI solution or suspension at its target drug loading. To ensure complete drug dissolution and homogeneity, sample aliquots (1–2 mg, *n* = 3) were collected from the drug formulation and dissolved in ACN and drug concentration was quantified by HPLC analysis.

### Scanning electron microscopy (SEM) imaging and SEM energy dispersive X-ray analysis (SEM-EDX)

Implant microstructures were evaluated by scanning electron microscopy (SEM) as previously described^20,30^. In brief, to assess the microstructure of in vitro ISFIs, 30 ± 3 mg of ISFI solution or suspension was injected into 200 mL of 0.01 M PBS, pH 7.4 with 2% Solutol at 37 °C. At predetermined time points (3-, 30-, and 90-days post incubation), the implants were removed from the bath solution, flash-frozen, and lyophilized for 48 h (FreeZone Benchtop Freeze Dryer, Labconco, Kansas City, MO). Sample preparation of ISFIs for SEM imaging before day 3 was not possible due to implant distortion likely caused from incomplete phase inversion and solvent exchange^20,27,53^. After freeze-drying, all depots were cut in half to expose the cross-section for imaging. The lyophilized samples were subsequently mounted on an aluminum stub using carbon tape, and sputter coated with 9 nm of gold-palladium alloy (60:40) (Hummer X Sputter Coater, Anatech USA, Union City, CA). The coated samples were then imaged using a Zeiss Supra 25 field emission scanning electron microscope with an acceleration voltage of 5 kV, 30 µm aperture, and average working distance of 10 mm (Carl Zeiss Microscopy, LLC, Thornwood, NY). ISFIs that underwent SEM energy dispersive X-ray (EDX) imaging/analysis were mounted on aluminum stubs using carbon tape, and sputter coated with 3 nm of gold conductive coating. The coated samples were then imaged using a Hitachi S-4700 Field Emission Scanning Electron Microscope at an acceleration voltage of 20 kV, 10 µA beam current, and average working distance of 12 nm. EDX analysis was performed on Oxford INCA PentaFet-X3 attached to the microscope at an acceleration voltage of 20 kV.

### In vitro cumulative drug release

In vitro release studies were performed similarly as previously described^20^. In brief, in vitro drug release kinetics of CAB ISFIs were investigated by injecting the drug-loaded polymer solution or suspension (30 ± 3 mg) into 200 mL of release medium (0.01 M PBS pH 7.4 with 2% Solutol) and incubating under sink conditions at 37 °C. The addition of Solutol increases CAB solubility in release media ensuring sink conditions are maintained^52^. The saturation solubility of CAB increased by 4.3-fold in PBS with 2% Solutol compared to PBS alone. Sink conditions were defined as the maximum CAB concentration in PBS with 2% Solutol being less than 1/5 times the saturation solution^52^. The release medium was completely removed and replaced with fresh medium every week to maintain sink conditions. Sample aliquots (1 mL) were collected daily for a week and weekly thereafter. The drug concentration in the release medium was determined by HPLC. Cumulative drug release was calculated from the HPLC analysis and was normalized by total mass of drug in the implant. All experiments were performed in triplicate.

### Stability studies

ISFI stability studies were similarly performed as previously described^20^. CAB ISFI formulations were stored at 4 °C or in a glass chamber at 40 °C/75% relative humidity (RH) in a Fisher Scientific Isotemp Incubator (Pittsburgh, PA). The glass chamber where the formulation vials were contained included a saturated salt aqueous solution of sodium chloride to achieve 75% relative humidity at 40 °C^54^. A humidity sensor was included within the glass chamber to verify 75% relative humidity. Formulations were removed 30- and 90-days post-incubation and sample aliquots (1–2 mg, n = 4) were collected and analyzed by HPLC to assess drug content and possible drug degradation. The formulations were also visually inspected for any change in their physical state (i.e., color and viscosity). A post-storage in vitro release study was performed if the formulation demonstrated physical stability (i.e., no discoloration, phase separation or precipitation), similar drug content to the control at time 0 (<10% difference), and a homogeneous suspension.

### Gel permeation chromatography (GPC)

GPC was performed as previously described^30^. In brief, GPC was utilized to evaluate polymer degradation of placebo ISFIs post-storage as well as polymer degradation in CAB ISFIs that were retrieved 30-, 60-, and 90-days post-injection from BALB/c mice. GPC analysis of placebo ISFIs, CAB ISFIs, and neat polymer was performed on Tosoh Biosciences EcoSEC Elite HLC-8420 equipped with a TSKgel GMH-M column. The column dimensions are 7.8 mm × 30 cm with a pore size of 5 microns. Tetrahydrofuran was used as the mobile phase at a flow rate of 0.5 mL/min. Molecular weight was reported relative to polystyrene standards and analyzed via refractive index (RI) detection.

### In vivo safety studies in BALB/c mice

A 30-day in vivo study was carried out to assess the systemic and local inflammation of CAB ISFIs in female BALB/c mice (8–10 weeks, Jackson Laboratory). All experiments were carried out with an approved protocol by the University of North Carolina Animal Care and Use Committee. Housing conditions for mice included a 12 h/12 h light/dark cycle at ambient temperature of 68–72°Fahrenheit with 30–70% humidity. Mice were subcutaneously injected with 50 µL of the CAB ISFI formulation with a 19 G needle (*n* = 9 mice). Two mice did not receive an injection and were used as the control. On day 3, 7, and 30 post-administration, mice were sacrificed (*n* = 3/timepoint for CAB ISFI treatment groups and *n* = 1 control mouse at day 3, and 30 that did not receive any treatment or injection) and blood samples via heart puncture were collected into capillary tubes and stored at −80 °C to quantify proinflammatory cytokines such as, tumor necrosis factor alpha (TNF-α) and interleukin-6 (IL-6) by enzyme-linked immunosorbent assay (ELISA, MAX™ Deluxe sets, BioLegend®). The ISFI was harvested for histology by circumferentially excising the depot and surrounding 1 cm of skin with subjacent subcutaneous adipose and placing the entire specimen flat on card stock to fix the tissue flat. The specimen was placed in 10% neutral buffered formalin at a ratio of 1:10 tissue:fixative at room temperature for 72 h, and then transferred to 70% ethanol at room temperature until specimen grossing. Fixed skin specimens were sectioned through the middle with the depot centrally oriented and the cut skin was embedded on-edge into paraffin blocks. Samples were sectioned at 4 µm and stained with Hematoxylin and Eosin using the autostainer XL from Leica Biosystems. The sections were stained with Hematoxylin (Richard-Allen Scientific, 7211) for 2 min and Eosin -Y (Richard-Allen Scientific, 7111) for 1 min. Clarifier 2 (7402) and Bluing (7111) solutions from Richard-Allen Scientific were used to differentiate the reaction. Inflammation was scored by a board-certified veterinary pathologist, as follows: 0: inflammatory cells present within expected limits; 1: minimal inflammation, few increased, scattered immune cells present; 2: mild inflammation, small clusters of immune cells to thin or localized tracks of inflammation or mild increase of the number of cells diffusely surrounding the depot; 3, moderate, thicker or multiple tracks of inflammation or moderate numbers of cells diffusely surrounding the depot; 4, severe, coalescing tracks of inflammation large enough to replace normal tissue architecture or severe numbers of cells diffusely surrounding the depot; 5, marked, inflammation present that is replacing expansive areas of normal tissue architecture. Local and systemic inflammation of treatment groups were compared to the no injection control group.

### In vivo pharmacokinetic studies in BALB/c mice

A 90-day in vivo study was conducted to assess in vivo pharmacokinetics of CAB ISFIs in female BALB/c mice (8–10 weeks, Jackson Laboratory). All experiments involving mice were carried out with an approved protocol by the University of North Carolina Animal Care and Use Committee. Housing conditions for mice included a 12 h/12 h light/dark cycle at ambient temperature of 68–72° Fahrenheit with 30-70% humidity. Mice were subcutaneously injected with 50 µL of the CAB ISFI formulation with a 19 G needle (*n* = 6 mice). At 1 h, 1 day, 3 day, 7 day, 30 day, 60 day, and 90 day, peripheral blood was collected from mice into capillary tubes coated with EDTA to isolate plasma. All samples were stored at −80 °C until PK analysis. CAB was extracted from mouse plasma by a liquid–liquid extraction followed by analysis by LC-MS/MS. Briefly, 25 μL of mouse plasma was mixed with 40 μL of 80:20 water:methanol containing the stable, isotopically-labeled ^13^C,^2^H_2_,^15^N-CAB and 300 μL of ethyl acetate. After mixing and centrifugation, the organic layer was removed and evaporated to dryness. The extracts were reconstituted with 75 μL of 75:25 water with 0.1% formic acid:acetonitrile with 0.1% formic acid prior to LC-MS/MS analysis. Chromatographic separation was performed on a Waters XTerra MS C18 (50 × 2.1 mm, 3.5 μm particle size) analytical column under gradient conditions with detection on an AB Sciex API-5000 triple quadruple mass spectrometer. The calibration range for the assay was 25–50,000 ng/mL. Calibration standards and quality control samples were within 15% of nominal concentrations.

### ISFI removal from BALB/c mice, biodegradation studies, and residual drug quantification

To assess the ability to safely remove an ISFI post administration in case of adverse events, CAB ISFIs were subcutaneously injected (50 µL) into female BALB/c mice (8–10 weeks, Jackson Laboratory) (*n* = 18) and ISFIs were removed at day 30 (*n* = 6), 60 (*n* = 6), and 90 (*n* = 6). All experiments involving mice were carried out with an approved protocol by the University of North Carolina Animal Care and Use Committee. Housing conditions for mice included a 12 h/12 h light/dark cycle at ambient temperature of 68–72° Fahrenheit with 30–70% humidity. Mice were euthanized and ISFIs were removed by making a small incision adjacent to the injection site at 30 (*n* = 6), 60 (*n* = 6), and 90-days (*n* = 6) post-administration. ISFIs extracted at day 30, 60, and 90 were further processed to assess mass loss, polymer molecular weight decrease by GPC analysis (*n* = 3/timepoint), and to quantify residual drug (*n* = 3/timepoint). To quantify residual drug concentration, excess tissue was carefully removed from the implants. Implants were placed in ACN to facilitate drug extraction and residual drug concentration was quantified by HPLC analysis.

### Preparation and injections in polyacrylamide hydrogels

We utilized polyacrylamide hydrogels to assess CAB ISFI injectability at a similar injection volume given to non-human primates (1 mL). Preparation of polyacrylamide hydrogels (40 mL) were adapted from previously reported protocols^36,37^. Briefly, a 40 mL hydrogel was prepared by combining 40 wt% of acrylamide aqueous solution (16 mL), 2 wt% bis-acrylamide aqueous solution (2 mL), and 22 mL of 0.01 M PBS pH 7.4. The mixture was cooled to 4 °C, and 10 wt% APS (2 mL) and 200 µL of TEMED were subsequently added and placed at 4 °C overnight to allow polymerization of the solution. 1 mL of placebo ISFI or CAB ISFI formulation was injected into the hydrogel with a 16, 18, or 19 G needle to assess injectability.

### Macaque procedures

All animal procedures were approved by the Centers for Disease Control and Prevention (CDC) Institutional Animal Care and Use Committee. Macaques were housed at CDC under the care of CDC veterinarians in accordance with the Guide for the Care and Use of Laboratory Animals. All procedures were performed under anesthesia and all efforts were made to minimize discomfort including providing appropriate housing conditions, dietary supplements and ample enrichment opportunities.

Female rhesus macaques received subcutaneous injections of CAB ISFI (500 mg/mL) at two different locations in the upper back (0.5–1 mL per site). Briefly, anesthetized animals were placed in ventral recumbency position and the semi-aqueous ISFI suspension loaded in a 3 cc syringe was administered in the subcutaneous space using a 16 G needle. Digital pressure was briefly applied over the administration site after the needle was removed.

To assess the feasibility of implant removal and to measure the drug tail, ISFIs that remained palpable were surgically retrieved from two macaques at week 12 (RA-1097 and RA-1093; 2 implants each) and from one animal at week 14 (RH-42012; 1 implant recovered). Animals were placed in ventral recumbency and the implant locations were confirmed through digital palpation. A surgical scalpel was used to make a single dermal incision (~1 in.) over each implant. The ISFI material was carefully separated from surrounding connective tissue and recovered mostly intact using tweezers. The incision site was visually inspected for abnormalities and subsequently flushed with sterile saline prior to being closed with Polydioxanone Sutures. Animals were monitored weekly by attending vets to assess surgical sites for proper wound healing and to ensure no signs of local infection.

### Pharmacokinetic analysis of CAB ISFIs in rhesus macaques

For the longitudinal PK evaluation, six female rhesus macaques received CAB ISFIs followed by drug level measurements in blood plasma and rectal and vaginal tissues. Five of the animals received two 1 mL injections (total of 1000 mg CAB) and one animal (RH-1080) received injection volumes of 1 and 0.5 mL (total of 750 mg CAB). CAB levels were measured weekly in plasma for up to 11 months and in tissues at weeks 4, 8, and 12 using a validated LC-MS method as previously described^55^. Rectal and vaginal biopsies were collected from macaques RH-42012, RA-1048, and RA-1080 at weeks 4, 8, and 12. The lower limit of quantification was 1 ng/mg and 10 ng/mL for biopsy and plasma, respectively.

### SHIV challenge study in rhesus macaques

We used a repeat exposure SHIV162P3 rectal challenge model that predicted the clinical efficacy of all currently approved PrEP regimens^56^. SHIV162P3 was obtained from the NIH AIDS Research and Reference Reagent Program (ARP-6526) and expanded in rhesus macaque PBMCs. The final challenge stock was diluted to 37 TCID_50_ and stored individually as 1 mL aliquots in liquid nitrogen and thawed prior to each rectal challenge. In this model, untreated control rhesus macaques exposed to 37 TCID_50_ of SHIV162p3 are infected after a median of two challenges^56,57^. Six female rhesus macaques were used in the study (10 years old; 7–11 kg). Four were implanted with CAB ISFIs and were exposed rectally to SHIV162p3 twice a week during periods when CAB concentrations were above the four times protein-adjusted IC_90_ (4× PA-IC_90_). Of these, two were challenged between weeks 4 and 8 post-implantation for a total of eight SHIV challenges each. The CAB ISFI depots were surgically removed from both animals at week 12 to measure the drug tail and monitored for SHIV infection during the drug washout period. The remaining two animals were challenged twice a week between weeks 14 through 18; one of these animals (RH-1048) received six additional challenges between weeks 25 and 28. Infection outcome of CAB treated animals in each group was compared to two untreated animals challenged concurrently with the same SHIV162p3 stock and dose. Blood was collected weekly during the challenges and the follow up phase for diagnostic testing. SHIV RNA in plasma was quantified by an RT-PCR assay with a sensitivity of 50 RNA copies/ml. Virus-specific serologic responses were measured using a synthetic peptide enzyme immunoassay assay (BioRad, Genetic Systems HIV-1/HIV-2, Redmond, WA)^56^. Animals were considered protected if seronegative and viral RNA negative during the SHIV challenges and a follow up period of 16 weeks.

### Safety and tolerability of CAB ISFI implants in rhesus macaques

Weekly wellness-checks were done weekly to assess the safety and tolerability of CAB ISFI implants. Physical examinations included monitoring animals’ weight, general health status, and thorough inspection of the area surrounding the implants for visible skin reactions (Supplementary Fig. 8). Erythema and edema were graded visually based on the Draize scale and assessed a scored from 0 (none reaction) to 4 (severe reaction)^58^.

During implant removal, two 3 mm surgical punch dermal biopsies were collected at the middle and cranial aspect of the implant location to measure drug levels and for histological assessment. One biopsy was weighed, and flash frozen for drug level testing and the other fixed in 10% formalin for histological examination. Biopsies stored in formalin were bisected, embedded, sectioned, and stained with hematoxylin and eosin (H&E). Sections were evaluated for the type and intensity of inflammation, as well as abundance of fibrosis, and presence of necrosis. Sections were blinded and evaluated independently by two veterinary pathologists. We used a semi-quantitative score based on ISO guidelines (ISO 10993-6:2007) for inflammatory cells including neutrophils, lymphocytes, plasma cells, macrophages, eosinophils and reactive fibroblasts within the dermis and subcutaneous tissues and fibroblasts. The distribution and severity of infiltration by each cell type was scored on a scale of 0–5, with 0 representing no cells present and 5 representing extensive infiltration by the specific cell type.

### CAB ISFI residual drug quantification in rhesus macaques

Each implant (left and right) was extracted from the tissue and then placed in ACN in a BSL2 + sterile environment. The sample was then removed from the sterile environment and placed on a shaking incubator. Sample aliquots (1 mL, *n* = 3) from the extraction solution were collected and analyzed by HPLC.

### CAB clearance estimation to determine input rates of long-acting formulations

To support estimation of CAB ISFI input rates in macaques, individual CAB clearance rates were estimated by NCA using the linear up-log down trapezoidal rule in Phoenix WinNonlin v8.3 for 4 animals with plasma concentrations collected over 98 to 329 days post ISFI administration (Supplementary Fig. 9). This estimation could not be performed for the 2 animals (RH-1093 and RH-1097) whose implants were removed at 84 days post administration. Therefore, AUC_INF_ was derived based on the equation $f=\left(\right.{C}_{{last}}+\left({kel}*{{AUC}}_{{last}}\right)/({kel}*{{AUC}}_{{INF}})$^59^ where *f* is the fraction of dose absorbed (i.e., loaded mass – residual mass) and kel was fixed to 0.173 hr^-1^ based on data reported in the new drug application^39^. Clearance was then calculated by ${Cl}=({Dose}*F)/({{AUC}}_{{INF}})$. All calculations assumed bioavailability (*F*) via the SQ route of administration was 100%, which is consistent with assumptions used for reported population PK models of the LA CAB IM injection^39^.

To support comparison of CAB ISFI vs CAB LA input rates in macaques, Quintessa Graph Grabber v2.0.2 software was used to extract PK data from a published concentration vs time graph of 9 macaques given two 50 mg/kg IM injections of CAB LA 6 days apart^38^. NCA was used as above to estimate individual animals’ clearance assuming a weight of 8 kg.

### Statistical analysis

Statistical analyses were performed in GraphPad Prism version 9.4 (GraphPad Software, Inc., La Jolla, CA, USA). To analyze differences in in vitro CAB release profiles when varying drug loading and PLGA molecular weight, a two-way ANOVA test and a Tukey’s multiple comparisons test was performed with respect to timepoint and formulation. To analyze differences in post-storage in vitro CAB release profiles compared to baseline, a two-way ANOVA test and a Tukey’s multiple comparisons test was performed with respect to timepoint and storage condition. A two-way ANOVA test and a Sidak’s multiple comparisons test was performed with respect to timepoint and TNF-α and IL-6 concentrations in plasma to analyze differences in vivo systemic inflammation with the no injection control group. For all statistical tests, a *P* value of <0.05 was considered significant (95% confidence level).

### Reporting summary

Further information on research design is available in the Nature Portfolio Reporting Summary linked to this article.

### Supplementary information

Reporting Summary

Supplementary Information

### Source data

Source Data

## Data Availability

Source data are provided with this paper underlying Fig. 1a–c, 2a, c, 3e, 7b–d as well as Supplementary Fig. 1a–c, and 2a, b. All other data is provided within the paper. Source data are provided with this paper.
