# Author Correction: Ultra-long-acting in-situ forming implants with cabotegravir protect female macaques against rectal SHIV infection

**DOI:** 10.1038/s41467-024-45552-0

**Published:** 2024-02-05

**Authors:** Isabella C. Young, Ivana Massud, Mackenzie L. Cottrell, Roopali Shrivastava, Panita Maturavongsadit, Alka Prasher, Andres Wong-Sam, Chuong Dinh, Tiancheng Edwards, Victoria Mrotz, James Mitchell, Josilene Nascimento Seixas, Aryani Pallerla, Allison Thorson, Amanda Schauer, Craig Sykes, Gabriela De la Cruz, Stephanie A. Montgomery, Angela D. M. Kashuba, Walid Heneine, Charles W. Dobard, Martina Kovarova, J. Victor Garcia, J. Gerardo Garcίa-Lerma, S. Rahima Benhabbour

**Affiliations:** 1 Division of Pharmacoengineering and Molecular Pharmaceutics, Eshelman School of Pharmacy, University of North Carolina at Chapel Hill, Chapel Hill, NC USA; 2 Laboratory Branch, Division of HIV Prevention, National Center for HIV/AIDS, Viral Hepatitis, STD, and TB Prevention, Centers for Disease Control and Prevention, Atlanta, GA USA; 3 Division of Pharmacotherapy and Experimental Therapeutics, Eshelman School of Pharmacy, University of North Carolina at Chapel Hill, Chapel Hill, NC USA; 4 Joint Department of Biomedical Engineering, North Carolina State University and The University of North Carolina at Chapel Hill, Chapel Hill, NC USA; 5 Comparative Medicine Branch, Division of Scientific Resources, National Center for Emerging and Zoonotic Infection Diseases, Centers for Disease Control and Prevention, Atlanta, GA USA; 6 Infectious Diseases Pathology Branch, Division of High-Consequence Pathogens and Pathology, National Center for Emerging and Zoonotic Infection Diseases, Centers for Disease Control and Prevention, Atlanta, GA USA; 7 Department of Psychology and Neuroscience, University of North Carolina at Chapel Hill, Chapel Hill, NC USA; 8 Pathology Services Core, Lineberger Comprehensive Cancer Center, University of North Carolina School of Medicine, Chapel Hill, NC USA; 9 International Center for the Advancement of Translational Science, Division of Infectious Diseases, Center for AIDS Research, University of North Carolina at Chapel Hill, Chapel Hill, NC USA

**Keywords:** Drug delivery, HIV infections, Antiviral agents, Rhesus macaque

Correction to: *Nature Communications* 10.1038/s41467-023-36330-5, published online 09 February 2023

The original version of this Article contained an error in the third and fourth sentence of the Methods section under heading ‘SHIV challenge study in rhesus macaques’, which incorrectly read ‘The final challenge stock was diluted to 10 TCID50 and stored individually as 1mL aliquots in liquid nitrogen and thawed prior to each rectal challenge. In this model, untreated control rhesus macaques exposed to 10 TCID50 of SHIV162p3 are infected after a median of two challenges^56,57^.’ The correct version now reads ‘The final challenge stock was diluted to 37 TCID50 and stored individually as 1mL aliquots in liquid nitrogen and thawed prior to each rectal challenge. In this model, untreated control rhesus macaques exposed to 37 TCID50 of SHIV162p3 are infected after a median of two challenges, consistent with previously used SHIV challenges^56, 57^.’

This has been corrected in both the PDF and HTML versions of the Article.

