# Supplementary material for: Ultra-long-acting in-situ forming implants with cabotegravir protect female macaques against rectal SHIV infection
Source: Nat Commun. 2023 Feb 9;14:708. doi: 10.1038/s41467-023-36330-5 (PMC9911691; doi:10.1038/s41467-023-36330-5)
Supplement: Supplementary file 1 — Reporting Summary [file 41467_2023_36330_MOESM1_ESM.pdf]

nature portfolio

Corresponding author(s): J. Gerardo Garcia-Lerma, S. Rahima Benhabbour

Last updated by author(s): 2023/1/10

Reporting Summary

Nature Portfolio wishes to improve the reproducibility of the work that we publish. This form provides structure for consistency and transparency in reporting. For further information on Nature Portfolio policies, see our Editorial Policies and the Editorial Policy Checklist.

Statistics

For all statistical analyses, confirm that the following items are present in the figure legend, table legend, main text, or Methods section.

n/a	Confirmed	
☐	☒	The exact sample size (*n*) for each experimental group/condition, given as a discrete number and unit of measurement
☒	☐	A statement on whether measurements were taken from distinct samples or whether the same sample was measured repeatedly
☐	☒	The statistical test(s) used AND whether they are one- or two-sided<br>*Only common tests should be described solely by name; describe more complex techniques in the Methods section.*
☐	☒	A description of all covariates tested
☐	☒	A description of any assumptions or corrections, such as tests of normality and adjustment for multiple comparisons
☐	☒	A full description of the statistical parameters including central tendency (e.g. means) or other basic estimates (e.g. regression coefficient) AND variation (e.g. standard deviation) or associated estimates of uncertainty (e.g. confidence intervals)
☐	☒	For null hypothesis testing, the test statistic (e.g. *F*, *t*, *r*) with confidence intervals, effect sizes, degrees of freedom and *P* value noted<br>*Give P values as exact values whenever suitable.*
☒	☐	For Bayesian analysis, information on the choice of priors and Markov chain Monte Carlo settings
☒	☐	For hierarchical and complex designs, identification of the appropriate level for tests and full reporting of outcomes
☒	☐	Estimates of effect sizes (e.g. Cohen's *d*, Pearson's *r*), indicating how they were calculated

Our web collection on statistics for biologists contains articles on many of the points above.

Software and code

Policy information about availability of computer code

Data collection	For vitro studies, Agilent OpenLab software (version C.01.08) was used for HPLC data collection.
Data analysis	For in vitro and in vivo mouse studies, Microsoft Excel (Version 2208) and GraphPad Prism (Version 9.4) was used for data analysis. GraphPad Prism (Version 9.4) was used for statistical analysis of in vitro release studies and in vivo mouse safety data. Phoenix WinNonlin version 8.3 was used for non-compartmental analysis. Quintessa Graph Grabber version 2.0.2 software was used to extract PK data from a published concentration vs time graph of 9 macaques given two 50 mg/kg IM injections of CAB LA 6 days apart.

For manuscripts utilizing custom algorithms or software that are central to the research but not yet described in published literature, software must be made available to editors and reviewers. We strongly encourage code deposition in a community repository (e.g. GitHub). See the Nature Portfolio guidelines for submitting code & software for further information.

Data

Policy information about availability of data

All manuscripts must include a data availability statement. This statement should provide the following information, where applicable:

- Accession codes, unique identifiers, or web links for publicly available datasets
- A description of any restrictions on data availability
- For clinical datasets or third party data, please ensure that the statement adheres to our policy

The data underlying Fig. 1a,b,c, Fig 2a, Fig 3e, Fig 7b-d as well as Supplementary Fig. 1a,b,c, and Supplementary Fig. 2a-b are available in the associated source data file. All other supporting the findings of this manuscript are available from the corresponding authors (J.G.G.L and S.R.B) upon reasonable request.

Human research participants

Policy information about studies involving human research participants and Sex and Gender in Research.

Reporting on sex and gender	N/A
Population characteristics	N/A
Recruitment	N/A
Ethics oversight	N/A

Note that full information on the approval of the study protocol must also be provided in the manuscript.

Field-specific reporting

Please select the one below that is the best fit for your research. If you are not sure, read the appropriate sections before making your selection.

☒ Life sciences ☐ Behavioural & social sciences ☐ Ecological, evolutionary & environmental sciences

For a reference copy of the document with all sections, see nature.com/documents/nr-reporting-summary-flat.pdf

Life sciences study design

All studies must disclose on these points even when the disclosure is negative.

Sample size	The number of macaques and mice per group was selected to account for potential variability in drug metabolism which may be due, but not limited, to differences in age, and weight. We did not conduct a power analysis to determine group sizes for the PK and efficacy studies
Data exclusions	No samples or animals were excluded from analysis.
Replication	All in vitro studies had n=3 replicates. In vivo mouse pharmacokinetic studies had n=6 mice and in vivo mouse safety studies had n=3 mice per time point. Macaque PK studies had n=3 macaques, efficacy studies had n=4 challenged macaques (n=2 CAB ISFI treated macaques challenged between 4-8 weeks and n=2 CAB ISFI treated macaques challenged between 14-18 weeks with n=1 untreated macaque as the control), and safety studies had n=6 CAB ISFI treated macaques for Draize scale analysis and n=3 CAB ISFI treated macaques and n=1 untreated macaque as a control for histology results. All n's in these experiments had produced similar results suggesting successful replication and reproducibility.
Randomization	No randomization was used to determine how samples/animals were allocated to experimental groups. Mice were allocated to each experimental group. Each experimental group had animals with similar age and weight. Method section/pg 36-38. Macaques in the study groups were not randomly assigned as we generally try to maintain social pairs with appropriate cage separation (G2 panels to allow limited physical/visual interactions) throughout the study. However, the average age and weights were similar between the groups. Methods section pg 41 line 828
Blinding	Blinding was used to analyze histology samples to determine inflammation scores. The board-certified pathologist was given numbered samples, but not the allocation of the samples to treatment groups. Samples from control mice and macaques (no treatment, baseline inflammation score) were unblinded to the pathologist. Methods section/pg 36-37 and 41-42. Blinding was not relevant in the treated macaques as they were all receiving the same dose of CAB ISFI. Blinding was not relevant for mice PK studies as they were all receiving the same dose of CAB ISFI. Blinding was not relevant for in vitro studies as we had to track the individual CAB release of each implant in vitro for accurate results (n=3).

Reporting for specific materials, systems and methods

We require information from authors about some types of materials, experimental systems and methods used in many studies. Here, indicate whether each material, system or method listed is relevant to your study. If you are not sure if a list item applies to your research, read the appropriate section before selecting a response.

Materials & experimental systems

n/a	Involved in the study
☒	☐ Antibodies
☒	☐ Eukaryotic cell lines
☒	☐ Palaeontology and archaeology
☐	☒ Animals and other organisms
☒	☐ Clinical data
☒	☐ Dual use research of concern

Methods

n/a	Involved in the study
☒	☐ ChIP-seq
☒	☐ Flow cytometry
☒	☐ MRI-based neuroimaging

Animals and other research organisms

Policy information about studies involving animals; ARRIVE guidelines recommended for reporting animal research, and Sex and Gender in Research

Laboratory animals	Female BALB/c mice (8-10 weeks old,obtained from Jackson Laboratories) and female rhesus macaques (10 years old). Housing conditions for mice included a 12h/12h light/dark cycle with an ambient temperature of 68-72 degrees Fahrenheit with 30-70% humidity (Methods page 36).
Wild animals	No wild animals were used in the study.
Reporting on sex	All animals used in this study were females. Justification for female animals: only female macaques were available for use at the time of the study. Female mice were used to keep the sex/gender between species consistent. Findings do not apply to only female animals as CAB ISFI would be indicated for females and males. Future studies will include male animals.
Field-collected samples	No field collected samples were used in the study.
Ethics oversight	All mouse protocols were approved by the Institutional Animal Care and Use Committee at UNC Chapel Hill (protocol number/IACUC ID 20-276). Mice were maintained by the Division of Comparative Medicine at UNC Chapel Hill. All macaques procedures were conducted at the Centers for Disease Control and Prevention (CDC) and performed under approved Institutional Animal Care and Use Committee (IACUC) protocols 3000- and 3002-DOBMONC and 3254MASMONC

Note that full information on the approval of the study protocol must also be provided in the manuscript.
