# Supplementary material for: Ultra-long-acting in-situ forming implants with cabotegravir protect female macaques against rectal SHIV infection
Source: Nat Commun. 2023 Feb 9;14:708. doi: 10.1038/s41467-023-36330-5 (PMC9911691; doi:10.1038/s41467-023-36330-5)
Supplement: Supplementary file 2 — Supplementary Information [file 41467_2023_36330_MOESM2_ESM.pdf]

SUPPLEMENTARY MATERIALS

Supplementary Table 1. CAB saturation concentration in various solvents and release media

Solvent	CAB Saturation Solubility (mg/mL)
NMP	131.70 ± 3.66
NMP:Gelucire 44/14 (9:1 w/w)	134.21 ± 3.81
NMP:DMSO (9:1 w/w)	144.49 ± 1.99
NMP:DMSO (1:1 w/w)	167.12 ± 12.04
NMP:DMSO (2:8 w/w)	52.25± 6.39
DMSO	38.00
(NMP:DMSO 1:1 w/w):Tween 20 (9:1 w/w)	106.61 ± 3.05
NMP: b-CD (6:4 w/w)	81.75± 2.35
NMP: HP- b-CD (6:4 w/w)	89.58± 5.49
NMP: HP- b-CD (9:1 w/w)	158.22 ± 1.12
PBS	0.014 ± 0.001
PBS + 2% Solutol	0.06 ± 0.002

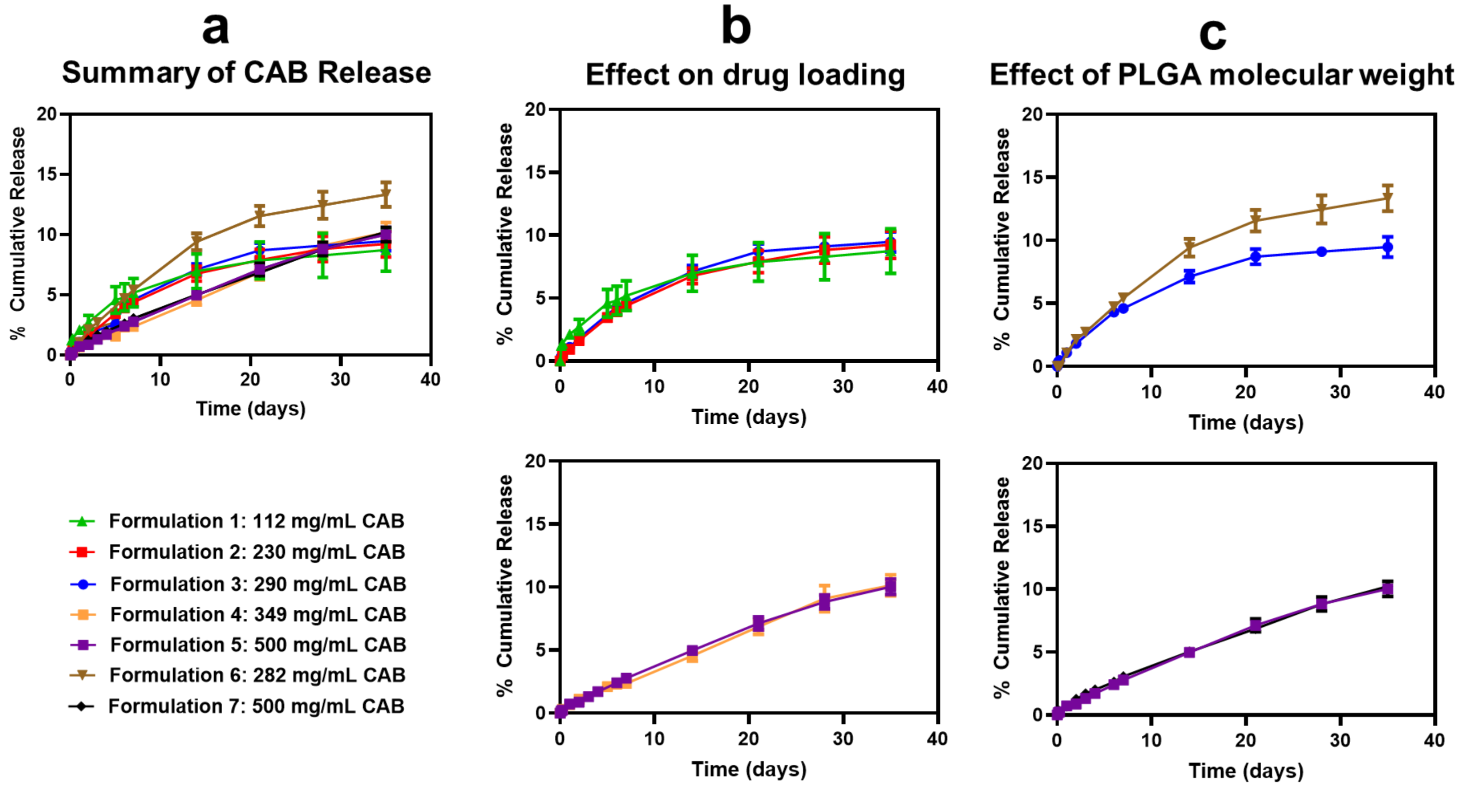

d

Formulation (F)	Analytical loading (mg/mL)	Burst at 24 hour (%)	Burst at 24 hour (µg)	% Cumulative Release at 35 days	Release rate (µg/day)	Projected duration to 100% release (days)
F1: 112 mg/mL 1:2 PLGA (27 kDa):Solvent	102.0 ± 5.92	2.12 ± 0.31	43.08 ± 2.96	8.74 ± 1.77	3.35	535
F2: 230 mg/mL 1:2 PLGA(27 kDa):Solvent	205.7 ± 2.78	0.93 ± 0.02	33.50 ± 2.63	9.24 ± 1.09	7.98	407
F3: 290 mg/mL 1:2 PLGA (27 kDa):Solvent	265.5 ± 5.81	1.09 ± 0.13	51.62 ± 6.94	9.47 ± 0.80	11.20	438
F4: 349 mg/mL 1:4 PLGA (27 kDa):Solvent	338.6 ± 21.57	0.68 ± 0.01	63.70 ± 3.20	10.19 ± 0.84	27.25	348
F5: 500 mg/mL 1:4 PLGA (27 kDa):Solvent	497.1 ± 22.96	0.73± 0.07	88.43 ± 10.07	10.03 ± 0.61	33.92	349
F6: 282 mg/mL 1:2 PLGA (10 kDa):Solvent	271.3 ± 0.99	1.08± 0.06	63.25 ± 3.63	13.34 ± 1.02	20.80	277
F7: 500 mg/mL 1:4 PLGA (10 kDa):Solvent	504.4 ± 14.60	0.69 ± 0.04	85.75± 9.41	10.22 ± 0.14	34.11	362

Supplementary Figure 1. CAB ISFI cumulative in vitro release kinetics. (a) % Cumulative release of all CAB ISFI formulations. (b) Effect of drug loading on % cumulative CAB release. (c) Effect of PLGA molecular weight on % cumulative CAB release. Data presented as average ± standard deviation for n=3 samples. (d) Summary table of release kinetics for CAB ISFI formulations. The solvent used in formulations is 1:1 (w/w) NMP:DMSO. Source data are provided as a Source Data file.

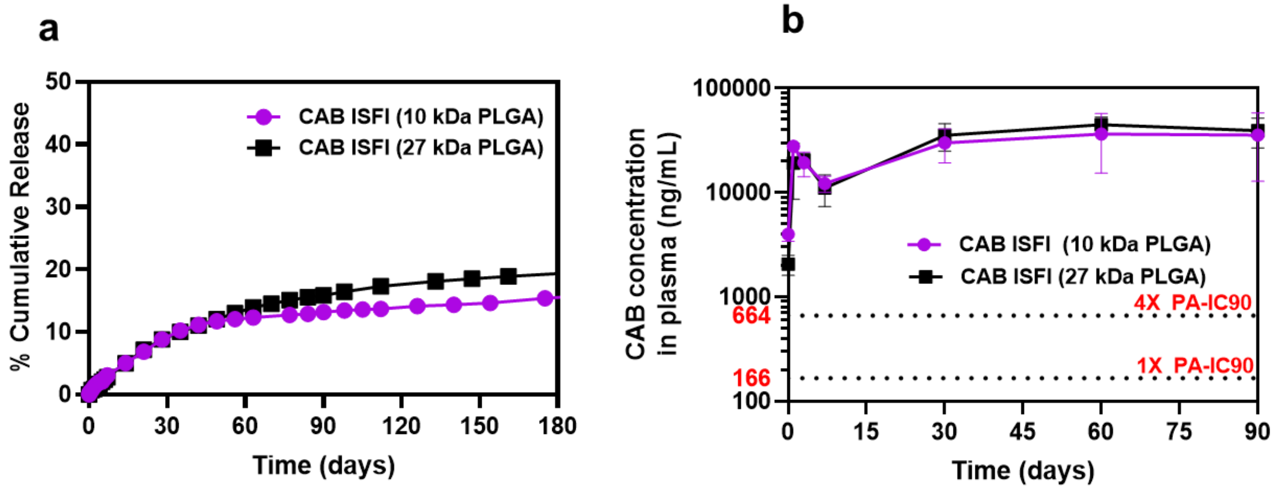

Supplementary Figure 2. Extended in vitro release and in vivo pharmacokinetics between Formulation 5 (500 mg/mL CAB 1:4 PLGA (27 kDa):solvent) and Formulation 7 (500 mg/mL CAB 1:4 PLGA (10 kDa):solvent). (a) % Cumulative release of CAB ISFI (10 kDa PLGA) (Formulation 7) and CAB ISFI (27 kDa) (Formulation 5) up to 180 days. In vitro release studies were done in phosphate buffer saline (PBS, pH 7.4 with 2% Solutol) at 37°C. Data are presented as average ± standard deviation for n=3 samples. (b) CAB concentration in plasma (average ± standard deviation) in female BALB/c mice (n=6/timepoint) after administration of CAB ISFI (10 kDa PLGA) (Formulation 7) and CAB ISFI (27 kDa) (Formulation 5). Source data are provided as a Source Data file.

Supplementary Table 2. Visocisty measurement of placebo ISFIs. The dynamic viscosity of placebo ISFIs (1:4 PLGA (27 kDa):solvent; Formulation 5 placebo and 1:4 PLGA (10 kDa):solvent; Formulation 7 placebo) was measured using a Brookfield Cone and Plate Digital Rheometer (Model: LVDV-III + CP, Middleboro MA, USA) at 25°C at a spindle speed of 2.70E-04 g and a shear rate of 3.84 1/s for 30 minutes. The temperature was maintained at 25°C using a circulating water bath (Brookfield TC-502, USA) surrounding an outer cylinder.

ISFI Formulation	Viscosity (cP)	Shear rate (1/s)	Spindle speed (g)	Torque (%)	Shear stress (D/cm^2)
1:4 w/w PLGA (27 kDa)/(1:1 NMP/DMSO) (placebo in Formulation 5)	79.3	3.84	2.70E-04	12.9	2.86
1:4 w/w PLGA (10 kDa)/(1:1 NMP/DMSO) (placebo in Formulation 7)	23.1	3.84	2.70E-04	4.3	0.94

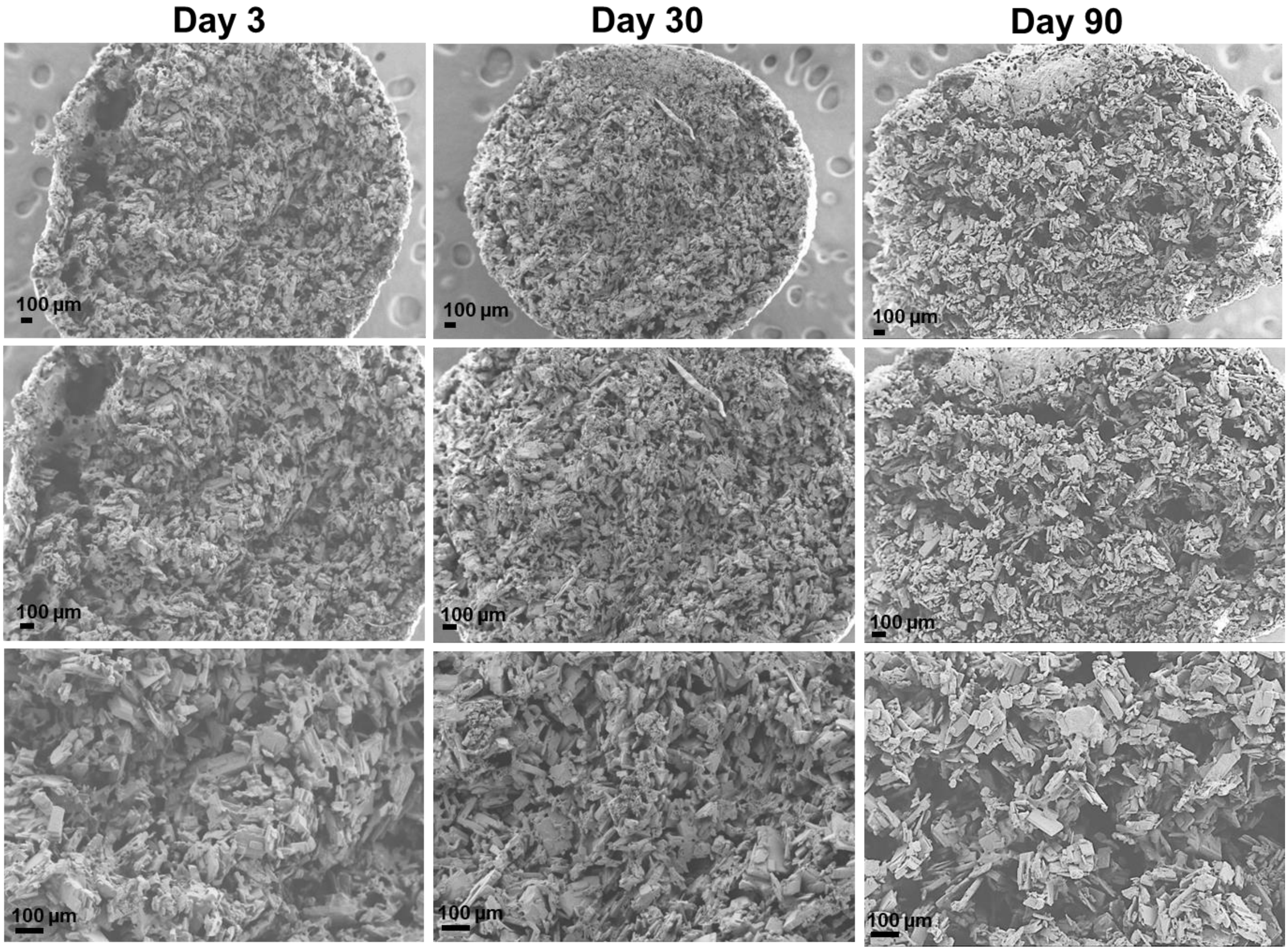

Supplementary Figure 3. CAB ISFI microstructure over time. SEM images of CAB ISFIs (500 mg/mL CAB (1:4 w/w PLGA (10 kDa): NMP/DMSO)) 3-, 30-, and 90-days post-incubation in vitro. Each column within the matrix represents increasing magnification (70X, 100X, and 200X). Scale bars represent 100 µm. Data was independently reproduced three times.

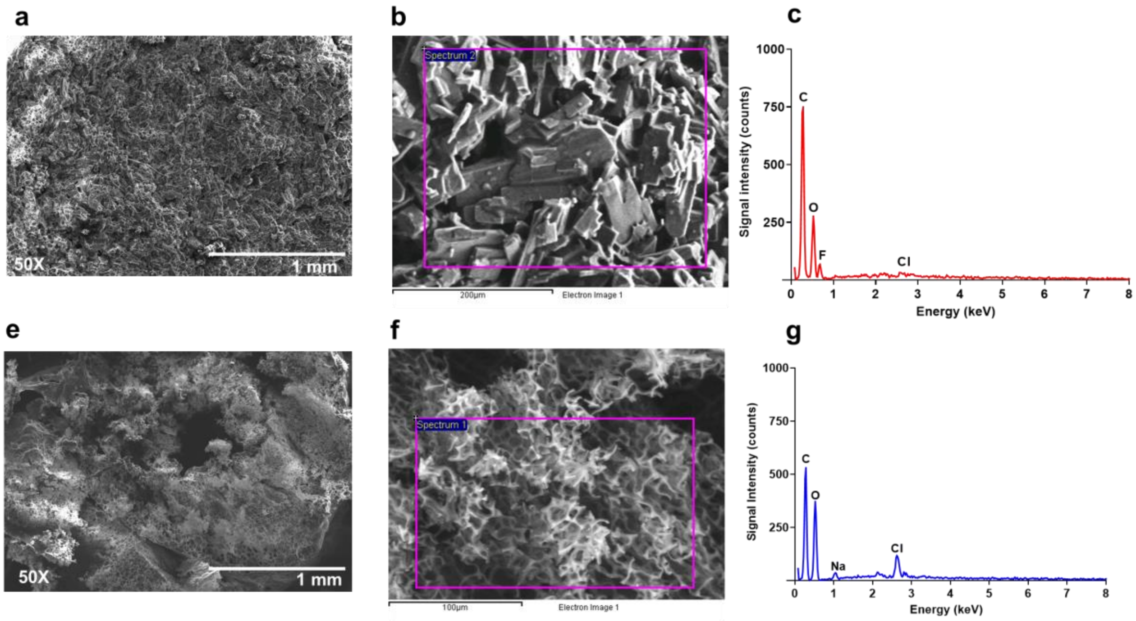

d

Element	Weight %	Atomic %
Carbon (C)	54.39	62.36
Oxygen (O)	34.44	29.64
Fluorine (F)	10.88	7.88
Chlorine (Cl)	0.29	0.11

h

Element	Weight %	Atomic %
Carbon (C)	50.27	58.25
Oxygen (O)	46.11	40.10
Sodium (Na)	1.10	0.67
Chlorine (Cl)	2.51	0.98

Supplementary Figure 4. Scanning electron microscopy (SEM) energy dispersive x-ray (EDX) of CAB ISFI and placebo ISFI (CAB-free). (a) SEM image of optimized CAB ISFI (scale bar = 1 mm), (b) SEM image of CAB ISFI (scale bar = 200 µm) used in EDX analysis (c) EDX analysis of CAB ISFI in (b) and (d). Summary table of EDX analysis of CAB ISFI representing carbon (C), oxygen (O), and fluorine (F) within the ISFI confirming crystals are composed of CAB. (e) SEM image of placebo ISFI (1:4 PLGA (10 kDa):solvent (CAB-free) (scale bar = 1 mm), (f) SEM image of placebo ISFI (scale bar = 200 µm) used in EDX analysis (g) EDX analysis of placebo ISFI in (g) and (h) Summary table of EDX analysis of placebo ISFI. No crystals or fluorine element are observed in the placebo ISFI confirming the crystals seen in (a) and (b) are CAB. Sodium (Na) and chloride (Cl) peaks are likely residual solvent from release media (PBS with 2% Solutol) during sample preparation. Data was independently reproduced two times.

a

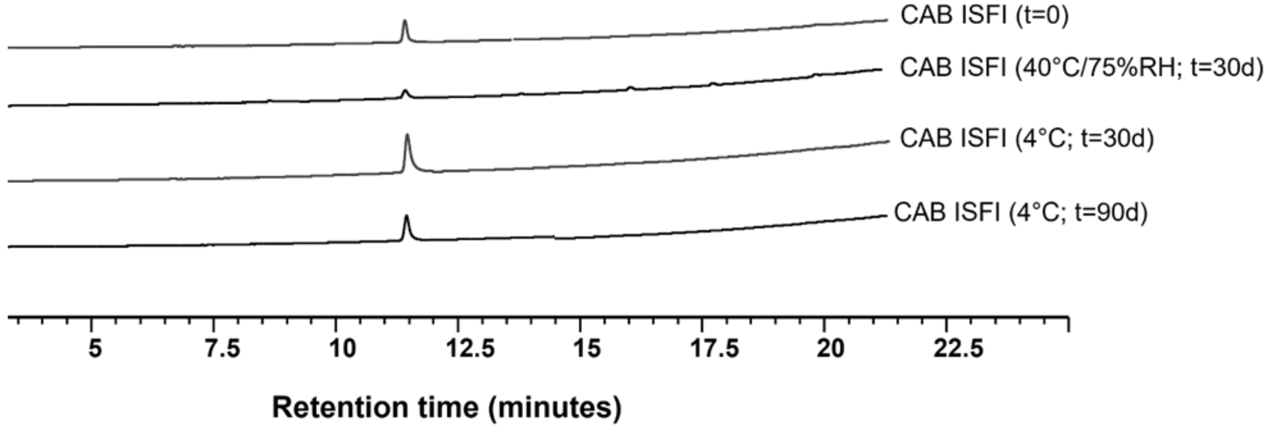

b

CAB concentration (mg/mL) at t=0 (baseline)	CAB concentration (mg/mL) at t=90 days in 40°C/75% RH
494.1±21.0	506.8±193.6

Supplementary Figure 5. Stability of CAB ISFIs. (a) HPLC chromatogram of CAB ISFI release samples at baseline (t=0) and after 30 days or 90 days in either 40°C/75% RH or 4°C storage conditions. HPLC drug peaks retention times were comparable. There were no degradation peaks in release samples. (b) CAB concentration in ISFI at baseline (t=0) compared to the concentration after 90 days of storage in 40°C/75% RH, indicating a non-homogenous suspension.

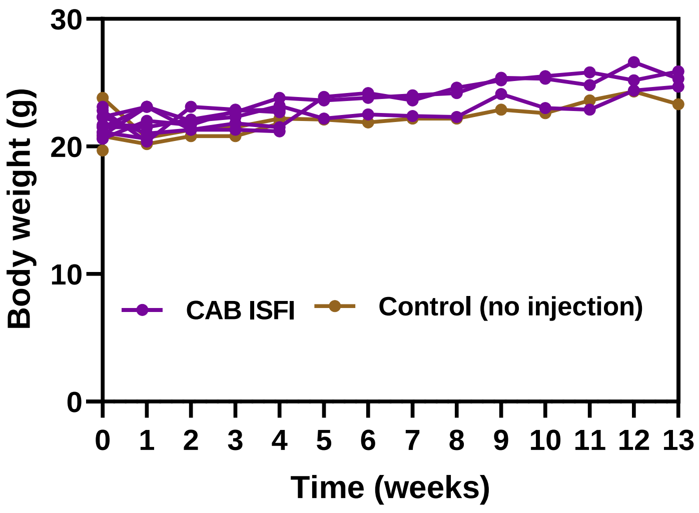

Supplementary Figure 6. Change in mouse body weight post-ISFI administration over 13 weeks. Mouse body weight for CAB ISFI (n=12) and for no injection control group (n=3). The data shows individual replicates for the no injection control group (n=3 from day 0-3, n=2 from day 3-week 4, n=1 from week 4-13). For the CAB ISFI treatment group, the data shows individual replicates (n=12 for day 0-3, n=9 for day 3-7, n=6 for week 1-4, n=3 for week 4-13). Statistical analysis: A two-way ANOVA test and a Sidak's multiple comparisons test was performed with respect to timepoint and mouse body weight to analyze changes in body weight when treated with CAB ISFI compared to the no injection control group. For all statistical tests, a P value of < 0.05 was considered significant (95% confidence level). Statistical analysis demonstrated no statistical significance ($p = 0.97$)

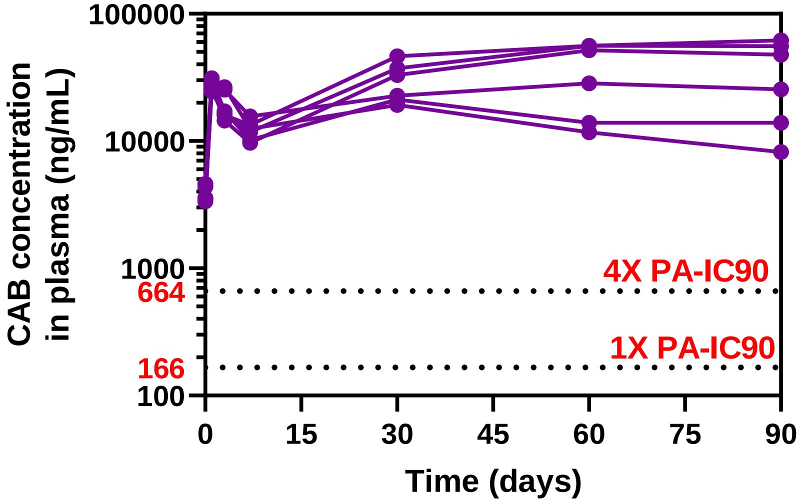

Supplementary Figure 7. CAB plasma concentrations plotted for individual mice. Plasma concentrations of CAB ISFI (1215 mg/kg, 50 µL injection; n=6-12/timepoint) for n=6 female BALB/c mice. 1× and 4× PA-IC90 values are indicated with dotted lines (166 ng/mL and 664 ng/mL).

Supplementary Table 3. Injectability of placebo ISFIs and optimized CAB ISFI formulation (1 mL injection volume) into polyacrylamide hydrogels.

Formulation	PLGA molecular weight	Needle Size	Injectable (Yes/No)?
Placebo: 1:4 w/w PLGA: (1:1 w/w NMP/DMSO)	10 kDa	19G	No
Placebo: 1:4 w/w PLGA: (1:1 w/w NMP/DMSO)	27 kDa	19G	Yes
Placebo: 1:2 w/w PLGA: (1:1 w/w NMP/DMSO)	27 kDa	19G	Yes
Placebo: 1:3 w/w PLGA: (1:1 w/w NMP/DMSO)	10 kDa	19G	Yes
500 mg/mL CAB (1:4 w/w PLGA: (1:1 w/w NMP/DMSO))	10 kDa	19G	No
		18G	Yes
		16G	Yes

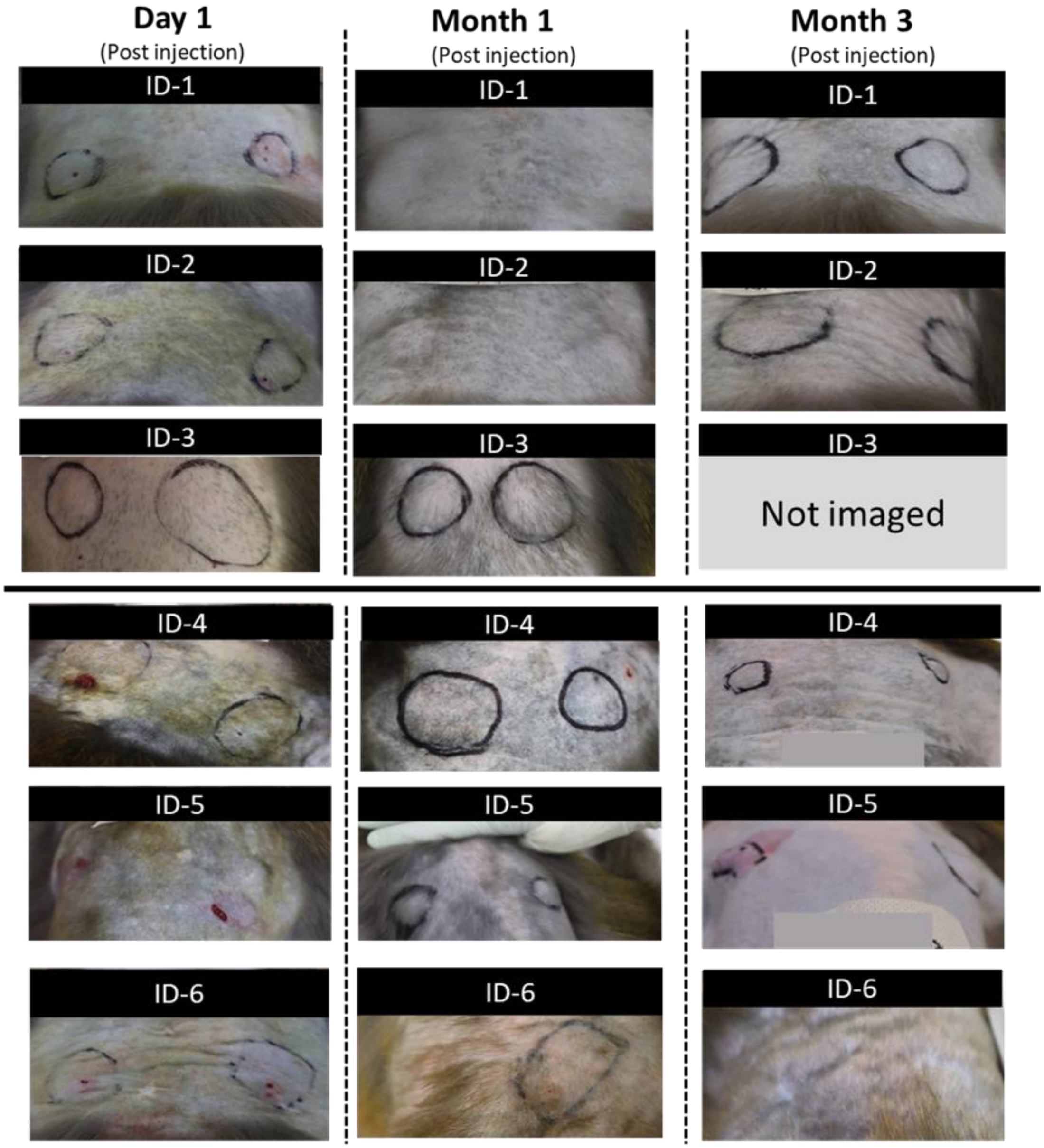

Supplementary Figure 8. **Longitudinal assessment of local skin reactions in rhesus macaques.** Visual inspection of injection sites in CAB ISFI treated macaques (n=6) were conducted weekly for up to 12 weeks. Images of injection sites were taken at day 1, month 1 and month 3 post injection.

Supplementary Table 4. Histopathological assessment in rhesus macaques. Subcutaneous tissue surrounding the implant site was collected 12 to 14 weeks post injection and scored (0 to 5 scale) for the presence of lymphocytes, plasma cells, histiocytes/macrophages, polymorphonuclear (PMN) cells, fibrosis, reactive fibroblasts (FB), and hemorrhage. Tissue collected in the scapular region from an untreated macaque (RH 44343) was used a control.

Animal ID	Lymph	Plasma cells	Histiocyte/ MP	MNGC	PMN	Fibrosis	Reactive FB	Hemorrhage	Neo-vascularization	Necrosis + type	Edema	Total Score
RH-44343 (untreated ctrl)	1	0	0	0	0	0	0	0	0	0	0	1
RH-1093 (CAB ISFI)	1	1	0	0	0	0	0	0	0	0	0	2
RH-1097 (CAB ISFI)	1	1	0	0	0	0	0	1	0	0	0	3
RH-42012 (CAB ISFI)	0	0	0	0	0	0	0	0	0	0	0	0

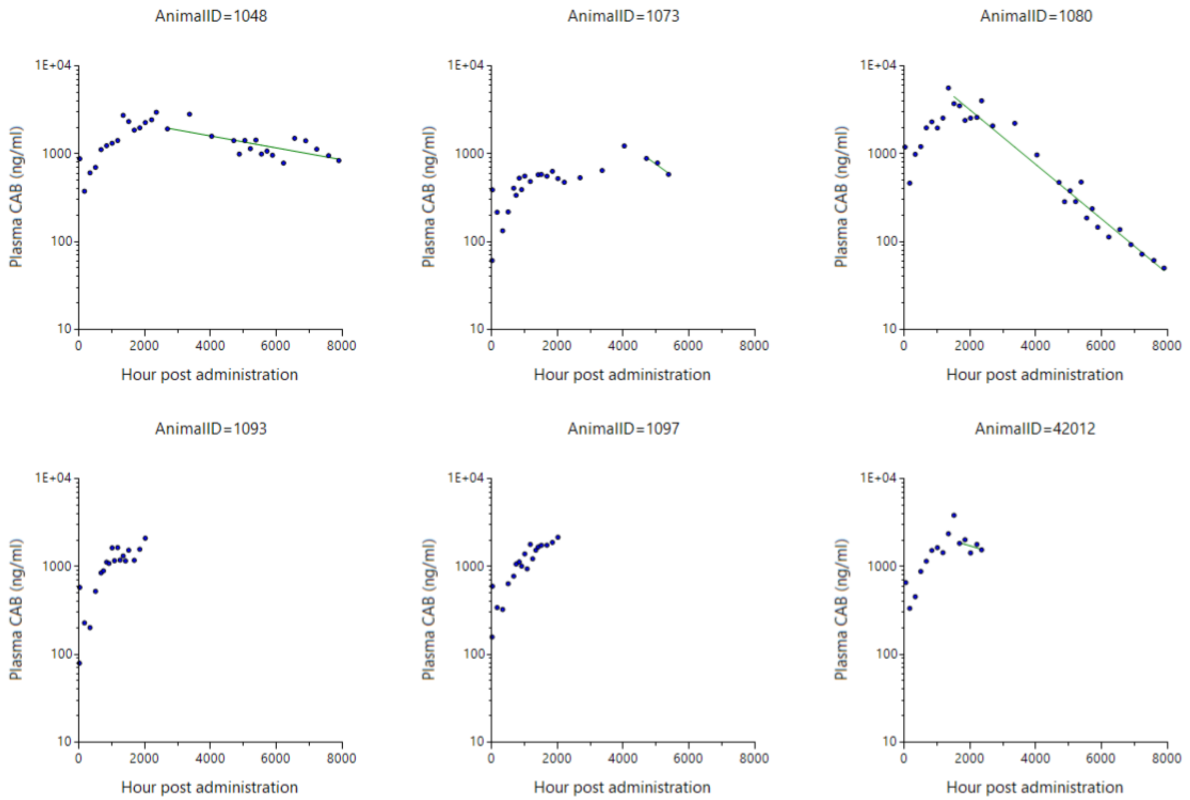

Macaque ID	Dose mg	Cmax ng/ml	Clast ng/ml	AUC_{last} $ng*hr*ml^{-1}$	AUC_{INF} $ng*hr*ml^{-1}$	Cl_{est} ml/hr	Cl_{calc} ml/hr
RH-1048	1000	2975	838	12211917	17749168	56	
RH-1073	1000	1230	583	3443367	4408201	227	
RH-1080	750	5628	50	10217635	10282758	73	
RH-1093	1000	2103	2103	2079535	Unable to estimate		287
RH-1097	1000	2153	2153	2360780	Unable to estimate		206
RH-42012	1000	3833	1550	3627136	9576078	104	
Cl_{est} was estimated from NCA of PK profile whereas Cl_{calc} was calculated based on residual CAB in the implants removed at day 84.							

Supplementary Figure 9. Noncompartmental analysis of individual macaques. Plasma concentration time profiles of 6 macaques dosed with 1.5-2 ml of CAB ISFI for noncompartmental analysis (NCA) with predicted values (green) overlaid on observed concentrations (blue). The linear up log down rule was used to calculate AUC.
